# Trends in Nutritional Biomarkers by Demographic Characteristics Across 14 Years Among US Adults

**DOI:** 10.3389/fnut.2021.737102

**Published:** 2022-01-13

**Authors:** Wenjie Wang, Fangzhou Zhu, Lanlan Wu, Shan Han, Xiaoyan Wu

**Affiliations:** ^1^Chronic Disease Research Institute, School of Public Health, School of Medicine, Zhejiang University, Hangzhou, China; ^2^Department of Nutrition and Food Hygiene, The National Key Discipline, School of Public Health, Harbin Medical University, Harbin, China; ^3^Department of Clinical Nutrition, Union Shenzhen Hospital of Huazhong University of Science and Technology, Shenzhen, China; ^4^Luohu Center for Disease Control and Prevention, Shenzhen, China; ^5^Department of Epidemiology and Biostatistics, School of Public Health, Guilin Medical University, Guilin, China

**Keywords:** nutritional biomarkers, nutritional status, temporal trend, US adults, demographic characteristics

## Abstract

**Background:** Understanding trend in nutritional status is crucial to inform national health priorities to improve diets and reduce related diseases. The present study aimed to analyze trends in the concentrations of all measured nutritional biomarkers and their status among US adults across 14 years.

**Methods:** Trends on the concentrations of nutritional biomarkers and nutritional status evaluated by the prevalence of deficiency, inadequacy, excess, and dyslipidemia were analyzed among US adults in 7 cross-sectional National Health and Nutrition Examination Surveys (NHANES 2003–2016) and by age, sex, race/ethnicity, and socioeconomic status.

**Results:** A total of 38,505 participants (weighted mean age of 47.2 years, 51.4% women) were included in the present study. Across 14 years, increased trends were found in red blood cell (RBC) folate, serum vitamin B_12_, vitamin D and albumin, the prevalence of iodine deficiency, vitamin B_6_ inadequacy, and hypophosphatemia, whereas decreased trends were observed in serum vitamin E, phosphorus, total calcium, total protein, apolipoprotein B (Apo B), low-density-lipoprotein cholesterol (LDL-C), triglyceride (TG), total cholesterol (TC), blood lead, cadmium, mercury, and the prevalence of vitamin C deficiency, vitamin D inadequacy, iodine excess, and dyslipidemia with elevated LDL-C, TC, TG, and lowered HDL/LDL. Non-Hispanic blacks (NHB) and participants with low socioeconomic status were accounted for the poor nutritional status of most biomarkers compared to their comparts.

**Conclusion:** Most nutritional biomarkers and their status were improved among US adults from 2003 to 2016, but some specific populations should be paid much attention to improve their nutritional status, especially for NHB and participants with low socioeconomic status.

## Introduction

Diet and nutritional status play an important role in the prevention and management of leading causes of death and non-communicable diseases (1). In the United States, dietary risks accounted for more than 500,000 deaths per year and more than 5% of risk-attributable of cardiovascular diseases (CVDs), neoplasms, diabetes, diet-related cancers, obesity, etc. (2). Understanding their trends is crucial to inform national health priorities to improve diets and reduce the risk of diet-related diseases.

Self-reported dietary data is one of the main methods for assessing dietary intake, however, subjective recall poses a great challenge for obtaining an accurate evaluation of diet and nutritional status (3). Besides dietary intake data, nutritional biomarkers would provide less error, more proximal, and objective assessment of diet and nutritional status reflecting a combination of dietary intakes and supplements consumption and thus, they were strongly recommended in nutritional epidemiology (4, 5). The trends of several nutritional biomarkers have been reported in some studies, such as blood folate (6), serum vitamin C (7), B_12_ (8), and urinary iodine (9) in the National Health and Nutrition Examination Surveys (NHANES 1988–2010, 1988–2004, 1988–2006, and 2001–2012, respectively), serum 25-hydroxyvitamin D [25(OH)D] in the NHANES (1998–2014) and Canadian Multicentre Osteoporosis Study (CaMos 1997–2007) (10, 11), blood lead, cadmium, and mercury in Korea NHANES (KNHANES 2005–2011) (12), serum lipid profiles in the National Center for Health Statistics (NCHS; 1998–2010), and Coronary Artery Risk Development in Young Adults (CARDIA, 1985–2011) study (13, 14). However, limited evidence is available on the trends of all possible measured nutritional biomarkers at the population level and specific subgroups, which would help to provide important guidance to improve the nutritional status of the American population.

In the present study, data from 7 consecutive cycles of the NHANES (2003–2016) were employed to analyze trends in the concentrations of a total of 24 nutritional biomarkers and their status including the prevalence of deficiencies, insufficiencies, or excesses among US adults.

## Materials and Methods

### Study Population

The NHANES is a program of studies designed to assess the health and nutritional status of adults and children in the US, which was conducted by the NCHS/Center for Disease Control and Prevention (CDC) (15). The survey is unique in that it combined interviews and physical examinations. The interview included demographic, socioeconomic, dietary, and health-related questions. In addition, a direct standardized physical examination, such as body measurements, phlebotomy, and urine collections, was carried out in a mobile examinations center (MEC) (16). The present analysis focused on data from 6 2-year survey cycles of NHANES (2003–2004, 2005–2006, 2007–2008, 2009–2010, 2011–2012, 2013–2014, and 2015–2016). After excluding subjects aged ≤ 20 years and pregnant women, a total of 38,505 subjects were examined in the present study. Additionally, the numbers of subjects for each measured nutritional biomarker are inconsistent, which are summarized in Supplementary Table S1.

All respondents provided their written informed consents. The NHANES protocols were approved by the NCHS Research Ethics Review Board.

### Laboratory Methods

Serum vitamin A, B_12_, C, E, 25(OH)D, folate [both serum and red blood cell (RBC)], calcium, iron-status indicators [iron, ferritin, transferrin saturation (TS), and erythrocyte protoporphyrin (EP)], potassium, sodium, phosphorous, total protein, albumin, apolipoprotein B (Apo B), triglyceride (TG), total cholesterol (TC), high-density lipoprotein cholesterol (HDL-C), low-density lipoprotein cholesterol (LDL-C), plasma vitamin B_6_, blood lead, cadmium, mercury, urine arsenic, and iodine were examined in the NHANES. Not all biomarkers were measured in each examined survey. The methods of assay for each biomarker and the survey years are described in Supplementary Table S2. As per NCHS recommendations, the fractional polynomial regression equation was applied to convert the Bio-Rad (BR) Quanta Phase II radio-assay blood folate results to equivalent values to match the microbiological assay (MA) blood folate (6). For serum 25(OH) D, calibrated harmonized data were used to adjust for differences in assay methodology as recommended by CDC using the liquid chromatography-tandem mass spectrometry (LC-MS/MS) equivalent data for correct interpretation of trends in 25(OH) D data (17). It is to be noted that serum 25(OH)D was considered as the best level of vitamin D since it can be a relatively comprehensive reflection of systemic vitamin D reserves from food and endogenous skin synthesis through sunlight, and the LC-MS/MS method in the present study was considered as the best way to get correct levels of it among the current laboratory methods (18, 19). For LDL-C, when the values of TC are less than or equal to 400 mg/dl, the Friedewald calculation was employed: LDL-C = TC – [HDL-C + TG/5] (20).

Blood samples were collected and stored using standardized methods for each survey, followed by subsequent processing and using appropriate laboratory methods to quantify the biomarkers. The NHANES quality assurance and quality control (QA/QC) protocols met the 1988 Clinical Laboratory Improvement Act mandates. QA activities prior to data collection included equipment calibration and laboratory staff training, and QC activities during the collection included automated software editing, data analysis, and analytical processing by technicians. The quality control coefficients of variation (CV) for each biomarker in survey years are shown in Supplementary Table S3.

### Assessment of Subgroup Variables

Race-ethnicity was divided into Mexican American (MA), non-Hispanic white (NHW), non-Hispanic black (NHB), and others (includes race/ethnicity other than MA, NHW, and NHB, including multiracial) based on the self-reported data of the participants on race and Hispanic origin.

The socioeconomic status was defined according to educational attainment (EA) and a poverty income ratio (PIR), participants were classified into high (more than 12 completed years of EA and PIR ≥ 3.5), low (less than 12 years of EA and a PIR < 1.30), and medium (others) (21).

Dietary supplements (yes/no, NHANES 2007–2016) were obtained through a family interview. Frequency, duration, and dose of dietary supplements were collected in the past 30 days. More detailed information on the type of supplements is described in Supplementary Table S4.

### Assessment of Nutritional Status

The reference concentrations or cutoff points for each nutrient were employed to evaluate nutritional biomarkers status, such as deficiency, insufficiency, excess, and dyslipidemia (Supplementary Table S5).

### Statistical Analysis

Sample weights, which accounted for planned oversampling of some groups and adjust for non-response and non-coverage to ensure nationally representative estimates, were incorporated in all analyses (22). Arithmetic means and SE were used to describe the mean of serum concentrations of vitamin A, 25(OH) D, potassium, sodium, phosphorus, total calcium, Apo B, HDL-C, LDL-C, and TC. Geometric means (SEs) were used to describe mean serum concentrations of vitamin B_6_, B_12_, C, E, folate, iron-status indicator, lead, cadmium, mercury, arsenic, iodine, and TC, since symmetric distributions for these biomarkers were obtained after logarithm transformation of their original skewed distributions. Percentages (SE) were calculated to estimate deficiency, insufficiency, or excess of biomarkers, and dyslipidemia. SEs used to calculate 95% CIs were estimated by using Taylor series linearization, a design-based approach that accounts for the sample design (23).

Linear trends and quadratic trends of the concentrations and percentages of deficiencies, inadequacies, excesses, and dyslipidemia for each nutritional biomarker were estimated using a linear regression model by treating survey year and the square of survey year as a continuous variable, respectively. General linear regression and ANOVA were employed to assess the variability in subgroups (age, sex, race/ethnicity, and socioeconomic status). Student-Newman-Keuls (SNK) was applied for pairwise comparison when statistically significant variability was observed.

All analyses were performed by using SPSS 24.0 (SPSS Inc., Chicago, IL, USA), and graphics were finished by using R project 3.5.3 (The R Foundation for Statistical Computing, Vienna, Austria). Two-sided *p* < 0.05 was considered to be statistically significant.

## Results

### Participant Characteristics

A total of 38,505 US adults (18,966 men, 19,536 women) were included in the present analysis. The basic characteristics of the participants are described in Table 1. From 2003 to 2016, the percentage of elderly adults aged ≥60 years was increased from 23.0 to 28.2% (*p*-linear trend = 0.001), while the percentage of adults aged 40–59 years was decreased from 39.0 to 36.2% (*p*-linear trend = 0.018). The proportion of NHW was declined from 72.2 to 64.0% (*p*-linear trend = 0.009), whereas other races/ethnicities was increased from 9.0 to 15.9% (*p*-linear trend < 0.001). In addition, the proportion of participants with high socioeconomic status was increased from 38.9 to 42.3% (*p*-linear trend = 0.038). But no evident changes were observed for other characteristics.

**Table 1:** Characteristics of study participants in the National Health and Nutrition Examination Survey (2003–2016)^a^.

**Characteristic**	**2003–2004 *N =* 4848**	**2005–2006** ***N =* 4687**	**2007–2008 *N =* 5888**	**2009–2010** ***N =* 6170**	**2011–2012 *N =* 5522**	**2013–2014** ***N =* 5722**	**2015–2016 *N =* 5668**	***P*-trend^**b**^**
**Age groups (y)**								
Aged 20–39 y	1,551 (38.1)	1,633 (36.9)	1,864 (37.0)	2,037 (36.5)	1,920 (35.8)	1,909 (35.7)	1,904 (35.6)	0.130
Aged 40–59 y	1,396 (39.0)	1,484 (39.7)	1,870 (39.1)	2,060 (38.6)	1,811 (38.2)	1,972 (37.2)	1,863 (36.2)	0.018
Aged ≥60 y	1,901 (23.0)	1,570 (23.4)	2,154 (23.8)	2,073 (24.9)	1,791 (26.0)	1,841 (27.1)	1,901 (28.2)	0.001
**Sex**								
Women	2,430 (51.4)	2,300 (51.0)	2,978 (51.4)	3,164 (51.4)	2,782 (51.7)	2,964 (51.5)	2,921 (51.5)	0.661
**Race**								
Non-Hispanic white	2,591 (72.2)	2,359 (72.1)	2,752 (69.7)	2,959 (68.1)	2,030 (66.5)	2,452 (65.9)	1,849 (64.0)	0.009
Non-Hispanic black	962 (11.1)	1,087 (11.5)	1,219 (11.3)	1,116 (11.4)	1,445 (11.5)	1,165 (11.4)	1,189 (11.4)	0.640
Mexican American	937 (7.7)	910 (7.8)	1,013 (8.2)	1,126 (8.5)	735 (8.7)	765 (9.2)	986 (8.8)	0.361
Others	358 (9.0)	331 (8.7)	904 (10.9)	969 (12.0)	1,312 (13.3)	1,340 (13.5)	1,644 (15.9)	<0.001
**Socioeconomic status** ^**b**^								
High	1,257 (38.9)	1,400 (42.3)	1,455 (41.2)	1,472 (39.6)	1,420 (39.5)	1,550 (39.2)	1,348 (42.3)	0.038
Medium	2,617 (53.3)	2,427 (50.9)	3,005 (49.9)	3,227 (52.2)	2,868 (51.7)	3,033 (53.0)	2,982 (51.0)	0.822
Low	619 (7.8)	553 (6.7)	837 (9.0)	798 (8.3)	696 (8.7)	655 (7.7)	652 (6.7)	0.732
**Use of supplements**								
Use	2,496 (53.5)	2,263 (53.6)	2,682 (48.9)	2,878 (49.5)	2,688 (51.6)	2,878 (53.7)	2,951 (55.9)	0.152
Not use	2,339 (46.4)	2,417 (46.3)	3,201 (51.0)	3287 (50.5)	2,830 (48.3)	2,842 (46.3)	2,715 (44.1)	0.152

a *Values are presented as mean (SE) for continuous variables and n (%) for categorical variables; race and ethnicity were self-reported. Percentages were adjusted for NHANES survey weights*.

b *Calculated by using the linear regression model*.

### Trends of Nutritional Biomarkers for Vitamins

From 2003 to 2016, the increased trends were observed for the concentrations of vitamin B_12_, 25(OH)D and RBC folate, being from 461.21 to 524.81 pg/ml (*p*-linear trend < 0.001), 62.40 to 69.59 nmol/L (*p*-linear trend < 0.001), and 467.84 to 507.69 ng/ml (*p*-linear trend = 0.017), while decreased trend was found for vitamin E, being from 29.29 to 27.37 μmol/L (*p*-linear trend < 0.001). The inverse U-shaped trend was found for the concentration of vitamin B_6_ (*p*-quadratic trend = 0.006). No statistically significant trends were found for other vitamins concentrations (Table 2).

**Table 2:** Trends of nutritional biomarkers among US adults in the National Health and Nutrition Examination Survey (2003–2016).

**Characteristics**	**2003–2004**	**2005–2006**	**2007–2008**	**2009–2010**	**2011–2012**	**2013–2014**	**2015–2016**	***P*-linear^**b**^**	***P-*quadratic**
**Vitamins**									
Vitamin A (umol/L)	2.10 (0.02)	2.13 (0.02)	—	—	—	—	—	0.312	—
Plasma vitamin B_6_ (nmol/L)^a^	43.78 (2.18)	50.21 (1.35)	51.71 (1.64)	48.87 (1.18)	—	—	—	0.724	0.006
Serum folate (ng/mL)^a^	17.32 (0.35)	17.57 (0.37)	16.58 (0.43)	16.01 (0.23)	17.27 (0.33)	17.43 (0.30)	16.60 (0.35)	0.334	0.103
RBC folate (ng/mL)^a^	467.84 (5.97)	497.28 (6.17)	502.11 (11.58)	468.92 (7.06)	471.52 (9.66)	511.33 (8.48)	507.69 (8.18)	0.017	0.310
Vitamin B_12_ (pg/mL)^a^	461.21 (8.72)	471.95 (7.69)	—	—	529.66 (6.27)	524.81 (4.40)	—	<0.001	0.201
Vitamin C (umol/L)^a^	42.70 (1.82)	44.57 (1.01)	—	—	—	—	—	0.363	—
25-hydroxyvitamin D (nmol/L)	62.40 (1.65)	60.64 (1.08)	67.11 (1.00)	67.59 (1.39)	70.69 (1.58)	69.59 (1.31)	—	<0.001	0.406
Vitamin E (umol/L)^a^	29.39 (0.46)	27.37 (0.31)	—	—	—	—	—	<0.001	—
**Minerals**									
EP (umol/L)^a^^,^^c^	0.98 (0.02)	0.99 (0.02)	—	—	—	—	—	0.623	—
Ferritin (ug/L)^a^^,^^c^	42.84 (1.35)	38.83 (1.34)	36.52 (1.01)	39.87 (0.97)	—	—	—	0.134	0.002
Iron (umol/L)^a^^,^^c^	13.08 (0.17)	12.68 (0.27)	—	—	—	—	—	0.182	—
TS (%)^a^^,^^c^	20.65 (0.33)	20.00 (0.38)	—	—	—	—	—	0.172	—
Blood lead (ug/dL)^a^	1.54 (0.04)	1.43 (0.02)	1.39 (0.04)	1.24 (0.03)	1.10 (0.03)	0.98 (0.04)	0.93 (0.03)	<0.001	0.063
Blood cadmium (ug/L)^a^	0.38 (0.01)	0.37 (0.01)	0.38 (0.02)	0.36 (0.01)	0.34 (0.01)	0.30 (0.01)	0.29 (0.01)	<0.001	0.002
Blood total mercury (ug/L)^a^	0.98 (0.07)	1.06 (0.08)	0.95 (0.05)	1.04 (0.04)	0.86 (0.04)	0.81 (0.03)	0.81 (0.03)	<0.001	0.117
Potassium (mmol/L)	4.01 (0.01)	3.97 (0.01)	3.98 (0.01)	3.99 (0.01)	3.94 (0.01)	4.02 (0.02)	3.96 (0.01)	0.323	0.171
Phosphorus (mmol/L)	1.23 (0.005)	1.23 (0.003)	1.22 (0.004)	1.21 (0.003)	1.21 (0.002)	1.24 (0.005)	1.19 (0.008)	0.001	0.487
Sodium (mmol/L)	139.17 (0.07)	138.99 (0.13)	139.23 (0.16)	139.36 (0.09)	138.85 (0.08)	139.78 (0.11)	138.70 (0.15)	0.694	0.052
Total calcium (mmo/L)	2.39 (0.003)	2.37 (0.004)	2.35 (0.007)	2.36 (0.003)	2.35 (0.003)	2.36 (0.003)	2.34 (0.002)	<0.001	0.006
Urinary iodine (ng/mL)^a^	142.13 (3.90)	149.38 (5.56)	155.02 (4.39)	137.66 (4.78)	129.00 (5.02)	128.20 (4.96)	124.71 (4.73)	0.001	0.019
Urinary total arsenic (ug/L)^a^	8.44 (0.63)	9.81 (0.72)	8.44 (0.62)	10.15 (0.77)	7.08 (0.53)	6.49 (0.49)	6.33 (0.46)	<0.001	0.003
**Protein**									
Total protein (g/L)	71.92 (0.23)	70.90 (0.22)	71.39 (0.13)	71.27 (0.18)	71.01 (0.21)	70.29 (0.20)	70.25 (0.22)	0.002	0.084
Albumin (g/L)	42.75 (0.12)	42.41 (0.13)	42.62 (0.06)	42.80 (0.13)	43.13 (0.09)	42.77 (0.08)	43.67 (0.12)	<0.001	0.001
**Lipids**									
Apo B (mg/dL)	—	101.13 (1.46)	93.27 (0.73)	90.77 (0.80)	90.06 (0.95)	90.15 (0.64)	93.79 (0.69)	<0.001	<0.001
HDL-C (mg/dL)	53.87 (0.39)	54.35 (0.34)	51.85 (0.51)	53.01 (0.43)	52.83 (0.51)	53.03 (0.29)	55.52 (0.75)	0.200	<0.001
LDL-C (mg/dL)	116.90 (1.10)	115.21 (1.24)	115.80 (0.80)	115.94 (0.92)	115.23 (0.97)	111.45 (0.89)	113.00 (1.03)	0.001	0.411
TC (mg/dL)	201.57 (0.73)	198.57 (0.78)	197.18 (0.81)	195.98 (0.91)	195.22 (0.98)	189.33 (0.85)	192.39 (1.33)	<0.001	0.348
TG (mg/dL)^a^	122.55 (2.40)	117.41 (1.94)	114.37 (2.23)	107.65 (1.62)	111.87 (3.31)	98.28 (2.52)	107.32 (1.12)	<0.001	0.107

*SE, Standard Error; EP, Erythrocyte Protoporphyrin; TS, Transferrin Saturation; Apo B, apolipoprotein B; HDL-C, High-Density Lipoprotein Cholesterol; LDL-C, Low-Density Lipoprotein Cholesterol; TC, Total Cholesterol; TG, Triglyceride. Nutrients were measured in serum unless otherwise stated*.

a *Geometric mean*.

b *Calculated by using the linear regression model*.

c *Among 4,909 women's aged 20–49 years*.

**Figure 1:**
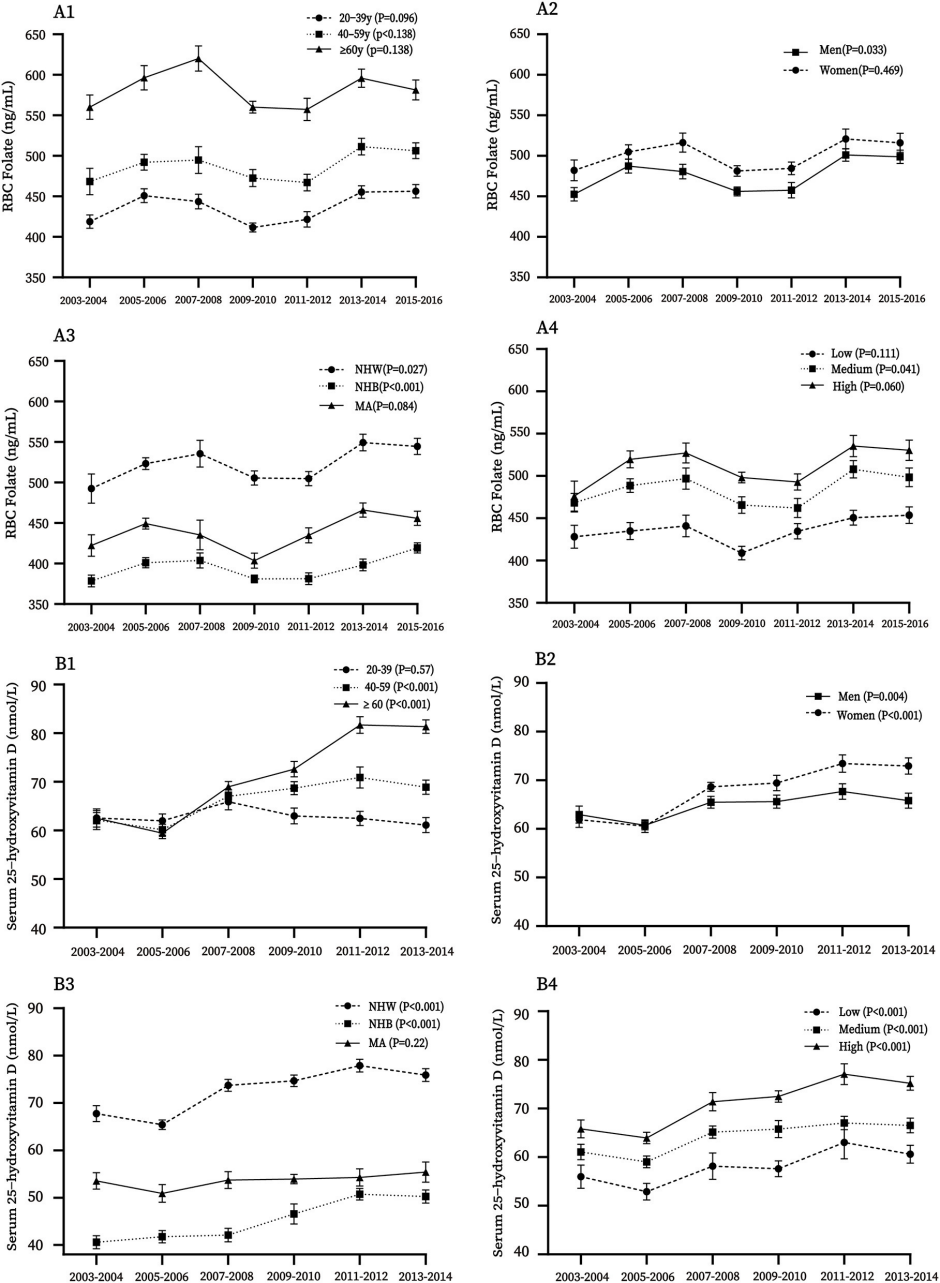
Trends of concentrations of RBC folate **(A1–A4)** and 25-hydroxyvitamin D **(B1–B4)** by age, sex, race-ethnicity, and socioeconomic status (SS) among US adults from 2003 to 2016. MA, Mexican American; NHW, non-Hispanic white; NHB non-Hispanic black.

In subgroup analysis, inverse U-shaped trends were observed in all subgroups except for participants aged ≥60 years, men, and participants with high socioeconomic status for plasma vitamin B_6_ (*p*-linear trend = 0.189, 0.569, and 0.199, respectively; Supplementary Figures S1A1–A4). The increased trends were observed in all subgroups for vitamin B_12_ (Supplementary Figures S1B1–B4), except for vitamin D among participants aged 40**–**59 years (*p*-linear trend = 0.57; Figures 1A1–A4), RBC folate among men, NHW, NHB, and participants with medium socioeconomic status (*p*-linear trend = 0.033, 0.027, <0.001, and 0.041, respectively; Figures 1B1–B4, Supplementary Table S6). Participants aged 20**–**39 years, NHB, and participants with low socioeconomic status for vitamin B_6_, folate, and 25(OH)D, men for serum and RBC folate, 25(OH)D, women for vitamin B_6_ were accounted for the lowest concentrations compared to their comparts, respectively (Supplementary Table S7).

In addition, the decreased trends were observed in the prevalence of vitamin C deficiency and vitamin D inadequacy, from 8.5 to 5.7% (*p*-linear trend = 0.032), and 22.9 to 18.8% (*p*-linear trend = 0.002), whereas an increased trend was found in the prevalence of vitamin B_6_ insufficiency, from 11.7 to 16.0 (*p*-linear trend = 0.005). The U-shaped trends were observed for the vitamin B_6_ and serum folate deficiencies (*p*-quadratic trend < 0.001 and = 0.005, respectively), and an inverse U-shaped trend was found for serum folate insufficiency (*p*-quadratic trend < 0.001). No statistically significant trends were observed for the prevalence of vitamin A, B_12_, D, and E deficiency, RBC folate deficiency, and insufficiency (*p*-linear trend = 0.834, 0.095, 0.335, 0.137, 0.059, and 0.141, respectively; Table 3).

**Table 3:** Trends of prevalence (SE) of deficiencies, insufficiencies, excesses, and dyslipidemia for nutritional biomarkers among US adults in the National Health and Nutrition Examination Survey (2003–2016)^a^.

**Characteristics**	**2003–2004**	**2005–2006**	**2007–2008**	**2009–2010**	**2011–2012**	**2013–2014**	**2015–2016**	***P*-trend^**b**^**	***P-*quadratic trend**
**Deficiencies (%)**									
Vitamin A	0.3 (0.1)	0.3 (0.1)	—	—	—	—	—	0.834	—
Vitamin B_6_	19.6 (1.5)	12.7 (0.9)	10.1 (0.9)	13.9 (0.9)	—	—	—	0.001	<0.001
Vitamin B_12_	2.0 (0.3)	1.9 (0.4)	—	—	1.7 (0.2)	1.8 (0.2)	—	0.095	0.231
Serum folate	0.6 (0.2)	0.2 (0.1)	0.0 (0.0)	0.2 (0.1)	0.1 (0.1)	0.1 (0.1)	0.2 (0.1)	0.055	0.005
RBC folate	1.0 (0.5)	0.6 (0.3)	0.1 (0.1)	0.3 (0.1)	0.3 (0.1)	0.3 (0.1)	0.2 (0.1)	0.059	0.189
Vitamin C	8.5 (1.1)	5.7 (0.7)	—	—	—	—	—	0.032	—
25-hydroxyvitamin D	7.7 (1.2)	5.2 (0.7)	6.4 (0.9)	6.9 (0.9)	5.6 (0.8)	5.8 (0.8)	—	0.335	0.738
Vitamin E	0.7 (0.2)	0.5 (0.1)	—	—	—	—	—	0.137	—
Iron	11.8 (1.3)	13.5 (0.7)	15.2 (0.9)	15.2 (1.3)	—	—	—	0.055	0.138
Iodine	31.2 (1.9)	31.4 (1.4)	30.1 (1.2)	33.9 (1.3)	36.9 (1.5)	40.7 (1.8)	41.8 (1.9)	<0.001	0.031
**Phosphorus**									
Hypophosphatemia	0.3 (0.1)	0.6 (0.1)	0.6 (0.1)	0.8 (0.1)	1.2 (0.2)	0.6 (0.1)	1.1 (0.1)	0.002	0.849
**Potassium**									
Hypokalemia	3.4 (0.3)	4.4 (0.2)	4.0 (0.4)	3.7 (0.4)	5.7 (0.7)	3.6 (0.3)	6.4 (0.5)	<0.001	0.023
**Sodium**									
Hyponatremia	1.5 (0.2)	2.5 (0.5)	2.6 (0.5)	1.8 (0.3)	3.2 (0.5)	1.9 (0.4)	2.3 (0.4)	0.376	0.081
Hypoproteinemia	0.4 (0.6)	0.9 (0.7)	0.5 (0.6)	0.6 (0.5)	1.0 (0.9)	1.5 (0.5)	1.6 (0.4)	0.008	0.125
Hypoalbuminemia	1.9 (0.2)	2.2 (0.1)	1.9 (0.1)	1.7 (0.1)	1.5 (0.2)	1.8 (0.2)	1.2 (0.2)	0.090	0.807
**Insufficiencies (%)**									
Vitamin B_6_	11.7 (1.3)	15.7 (0.8)	15.3 (0.6)	16.0 (0.5)	—	—	—	0.005	0.053
Serum folate	1.7 (0.6)	2.3 (0.6)	4.2 (0.5)	4.5 (0.4)	2.1 (0.4)	2.2 (0.2)	3.2 (0.3)	0.239	<0.001
RBC folate	0.1 (0.6)	0.0 (0.4)	0.3 (0.1)	0.1 (0.0)	0.3 (0.1)	0.3 (0.1)	0.1 (0.1)	0.141	0.320
25-hydroxyvitamin D	22.9 (2.0)	23.9 (1.8)	19.8 (1.2)	18.9 (1.2)	19.0 (1.9)	18.8 (1.2)	—	0.002	0.573
**Excesses (%)**									
Iodine	36.6 (1.3)	37.2 (1.0)	37.9 (0.8)	32.4 (1.7)	31.8 (1.9)	29.1 (1.9)	27.6 (1.8)	<0.001	0.101
**Potassium**									
Hyperkalemia	0.8 (0.1)	0.2 (0.1)	0.3 (0.1)	0.5 (0.1)	0.6 (0.2)	0.9 (0.3)	0.6 (0.2)	<0.001	0.023
**Sodium**									
Hypernatremia	0.4 (0.1)	0.3 (0.1)	0.9 (0.3)	0.5 (0.1)	0.4 (0.1)	1.2 (0.2)	0.2 (0.1)	0.198	0.005
Elevated blood lead	3.4 (0.2)	2.7 (0.3)	2.9 (0.3)	2.1 (0.3)	2.2 (0.6)	1.7 (0.5)	1.2 (0.2)	<0.001	0.697
Elevated blood cadmium	15.2 (0.7)	13.1 (0.9)	12.8 (1.1)	11.8 (0.7)	12.4 (0.9)	8.8 (1.1)	9.5 (0.9)	<0.001	0.996
Elevated blood mercury	11.2 (1.3)	10.7 (0.8)	9.6 (1.3)	12.0 (0.8)	9.5 (1.6)	8.6 (0.8)	7.9 (1.1)	<0.001	0.583
**Dyslipidemia**									
Elevated Apo B	—	12.8 (1.5)	6.5 (0.5)	5.9 (0.7)	4.8 (0.8)	6.0 (0.6)	5.1 (1.1)	0.047	<0.001
Lowered HDL-C	28.0 (1.0)	26.0 (1.0)	33.8 (1.3)	32.6 (0.9)	28.9 (1.7)	31.1 (1.2)	28.8 (1.4)	0.269	0.003
Elevated LDL-C	31.8 (1.5)	31.5 (1.5)	30.1 (1.0)	30.3 (1.2)	31.1 (1.1)	26.9 (1.2)	29.4 (1.0)	0.022	0.936
Lowered HDL-C/LDL-C	38.6 (1.6)	34.1 (1.4)	37.1 (1.2)	37.2 (1.2)	36.1 (1.5)	32.2 (1.5)	32.6 (1.4)	0.003	0.316
Elevated TC	47.5 (1.3)	44.2 (1.0)	43.1 (1.0)	42.3 (1.1)	42.3 (0.9)	36.0 (1.3)	38.8 (1.4)	<0.001	0.619
Elevated TG	33.1 (1.5)	30.1 (1.3)	30.2 (1.0)	25.0 (1.1)	26.5 (2.0)	23.3 (1.2)	22.0 (1.0)	<0.001	0.681

*SE, Standard Error; Apo B, apolipoprotein B; HDL-C, High-Density Lipoprotein Cholesterol; LDL-C, Low-density Lipoprotein Cholesterol; TC, Total Cholesterol; TG, Triglyceride*.

a *Serum measurement otherwise stated*.

b *Calculated by using the linear regression model*.

### Trends of Nutritional Biomarkers for Minerals

From 2003 to 2016, decreased trends were apparent in the concentrations of serum calcium, phosphorus, blood lead, cadmium, and mercury, from 2.39 to 2.34 mmo/L (*p*-linear trend < 0.001), 1.23 to 1.19 mmo/L (*p*-linear trend = 0.001), 1.54 to 0.93 μg/dl (*p*-linear trend < 0.001), 0.38 to 0.29 μg/dl (*p*-linear trend < 0.001), and 0.98 to 0.81 μg/dl (*p*-linear trend < 0.001), whereas no statistically significant trends were found for the concentrations of potassium and sodium (*p*-linear trend = 0.323 and 0.694, respectively). The inverse U-shaped trends were found for the concentrations of ferritin, urinary iodine, and total arsenic (*p*-quadratic trend = 0.002, 0.019, and 0.003, respectively; Table 2).

**Figure 2:**
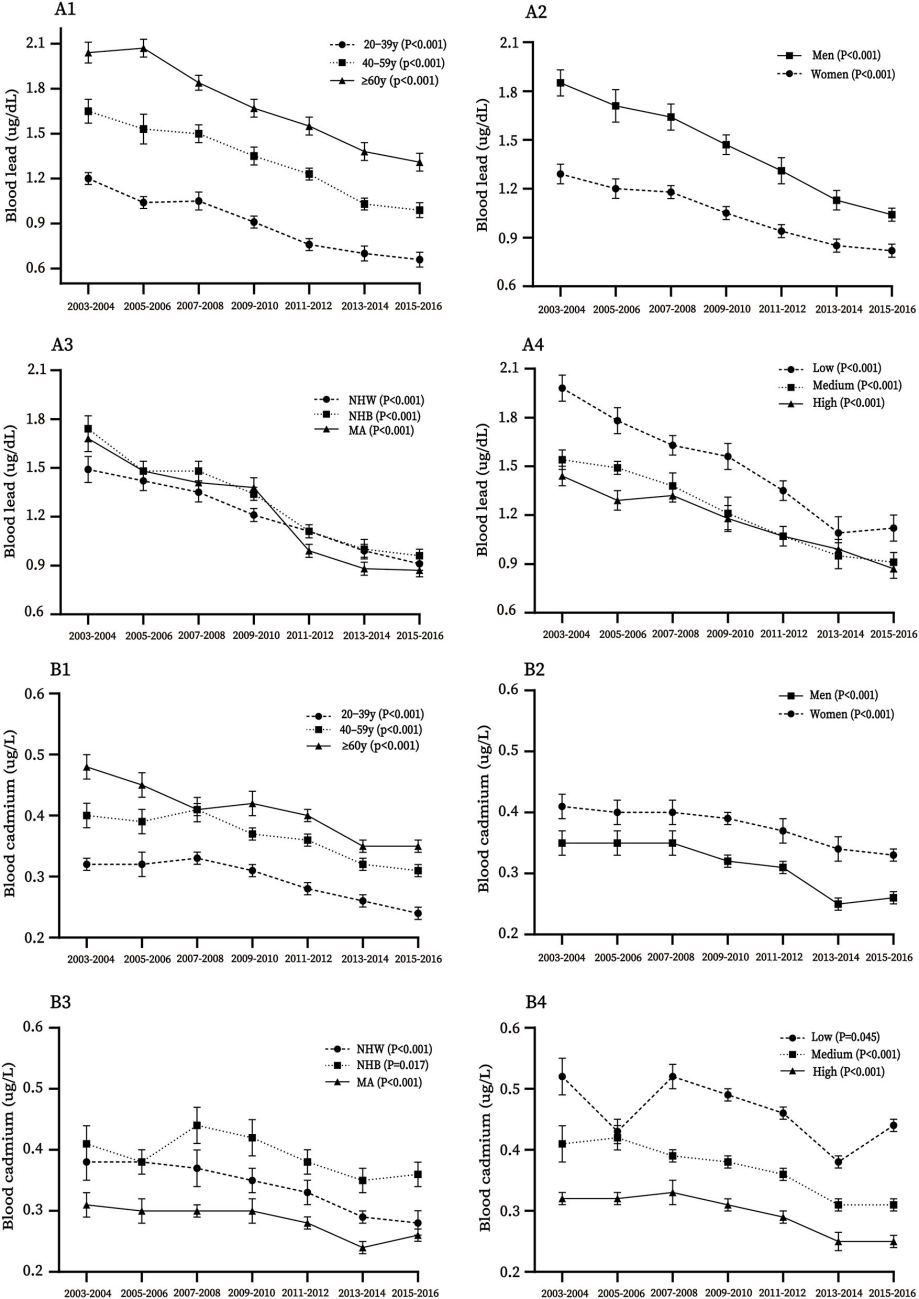
Trends of concentrations of blood lead **(A1–A4)** and cadmium **(B1–B4)** by age, sex, race-ethnicity, and socioeconomic status (SS) among US adults from 2003 to 2016. MA, Mexican American; NHW, non-Hispanic white; NHB, non-Hispanic black.

In subgroup analysis, the decreased trends were found in all subgroups for blood lead (Figures 2A1–A4), cadmium (Figures 2B1–B4), serum calcium (Supplementary Figures S2A1–A4), and phosphorus (Supplementary Figures S2B1–B4; all *p*-linear trend < 0.05) and were not found among participants aged ≥ 60 years, Mexican American, and participants with low socioeconomic status for blood mercury (*p*-linear trend = 0.263, 0.134, and 0.437, respectively) (Supplementary Figures S3A1–A4). Inverse U-shaped trends were found in all subgroups for urinary arsenic (all *p*-linear trend < 0.05; Supplementary Figures S3B1–B4), except for NHB for urinary iodine (*p*-linear trend = 0.84; Supplementary Figures S4A1–A4). In addition, participants aged ≥ 60 years, non-Hispanic blacks, and participants with low socioeconomic status for blood lead, cadmium, and mercury, women for blood cadmium, and men for blood lead and mercury accounted for the highest concentrations compared with their comparts. For urinary iodine, participants aged 40**–**59 years, women, NHB, and participants with high socioeconomic status accounted for the lowest concentrations compared with their comparts, respectively. The concentrations of nutritional biomarkers for each mineral by demographic variables are described in Supplementary Table S7.

In addition, the decreased trends were found for iodine excess, hyperkalemia, elevated lead, cadmium, and mercury, from 36.6 to 27.6% (*p*-linear trend < 0.001), 0.8 to 0.6% (*p*-linear trend < 0.001), 3.4 to 1.7% (*p*-linear trend < 0.001), 15.2 to 9.5% (*p*-linear trend < 0.001), and 11.2 to 7.9% (*p*-linear trend < 0.001), while the increased trends were observed for the prevalence of iodine deficiency and hypophosphatemia, from 31.2 to 41.8% (*p*-linear trend < 0.001) and 0.3 to 1.1% (*p*-linear trend = 0.002). The inverse U-shaped trends were observed for hypokalemia and hypernatremia (*p*-quadratic trend = 0.023 and 0.005, respectively; Table 3).

### Trends of Nutritional Biomarkers for Protein and Lipids

**Figure 3:**
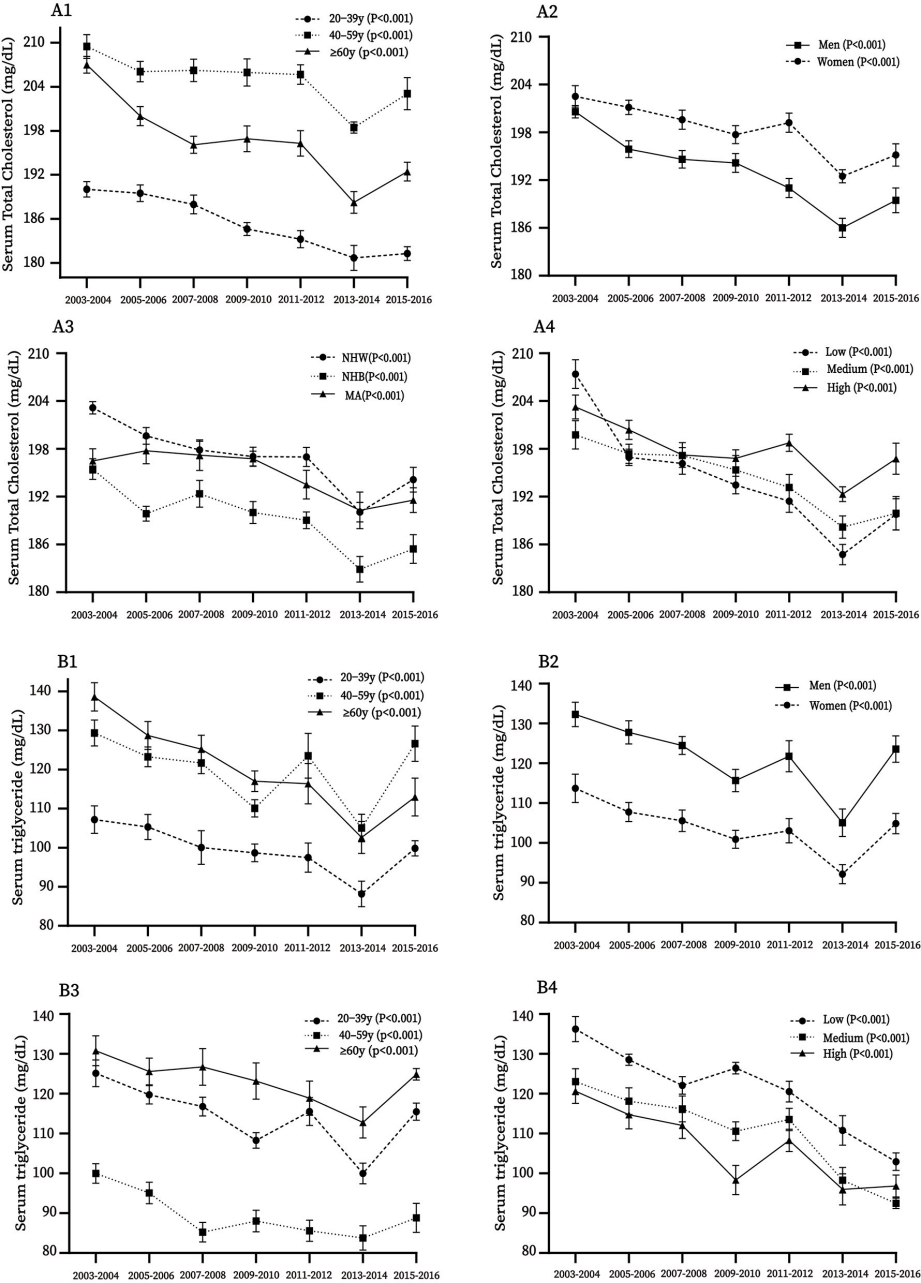
Trends of concentrations of serum total cholesterol **(A1–A4)** and triglyceride **(B1–B4)**, by age, sex, race-ethnicity, and socioeconomic status (SS) among US adults from 2003 to 2016. MA, Mexican American; NHW, non-Hispanic white; NHB non-Hispanic black.

Across 14 years, trends of serum total protein, Apo B, TC, TG, LDL-C were decreased from 71.92 to 70.25 g/L (*p*-linear trend = 0.001), 101.13 to 93.79 mg/dl (*p*-linear trend < 0.001), 201.57 to 192.39 mg/dl (*p*-linear trend < 0.001), 122.55 to 107.32 mg/dl (*p*-linear trend < 0.001), and 116.90 to 113.00 mg/dl (*p*-linear trend = 0.001), while serum albumin concentration increased from 42.75 to 43.67 g/L (*p*-linear trend < 0.001), respectively. The U-shaped trend was found in the serum LDL-C concentration (*p*-quadratic trend < 0.001; Table 2). In subgroups analysis, the decreased trends were observed in all subgroups for serum Apo B (Supplementary Figures S4B1–B4), TC (Figures 3A1–A4) and TG (Figures 3B1–B4; all *p*-linear trend < 0.05), except for participants aged 20**–**39 years and participants with high socioeconomic status for serum total protein (*p*-linear trend = 0.133 and 0.202, respectively; Supplementary Figures S5A1–A4). In addition, statistically significant linear trends were found in participants aged ≥ 60 years, men, NHW, and participants with medium socioeconomic status for LDL-C (*p*-linear trend < 0.001, = 0.001, 0.002, and 0.010, respectively (Supplementary Figures S6A1–A4).

The increased trends were observed in all subgroups for albumin (all *p*-linear trend < 0.05; Supplementary Figures S5B1–B4), but the U-shaped trends were not found in participants aged 40**–**59 years and Mexican American for HDL-C (*p*-quadratic trend = 0.157 and 0.192, respectively; Supplementary Figures S6B1–B4, Supplementary Table S6). Participants aged ≥60 years, women for serum total protein and albumin, NHW, and participants with high socioeconomic status for total protein, NHW, and participants with low socioeconomic status for albumin, participants aged 20**–**39 years, men, Mexican American, and participants with low socioeconomic status for HDL-C accounted for the lowest concentrations compared with comparts, respectively. Participants aged 40**–**59 years and Mexican American for Apo B, LDL-C, TC, and TG, men for Apo B, TC, and TG, participants with high socioeconomic status for LDL-C and TC, participants with low socioeconomic status for Apo B and TG accounted for the highest concentrations compared with comparts, respectively (Supplementary Table S7).

In addition, there were significantly decreased trends for the prevalence of dyslipidemia with elevated LDL-C, TC, TG, and lowered HDL-C/LDL-C from 31.8 to 29.4% (*p*-linear trend = 0.022), 47.5 to 38.8% (*p*-linear trend < 0.001), 33.1 to 22.0% (*p*-linear trend < 0.001), and 38.6 to 32.6% (*p*-linear trend = 0.003), but increased trend for hypoproteinemia being from 0.4 to 1.6 (*p*-linear trend = 0.008). The inverse U-shaped trends were found for the prevalence of elevated Apo B and lowered HDL-C, respectively (*p*-quadratic trend < 0.001 and = 0.003; Table 3).

### Sensitivity Analysis

When the trends analysis was conducted among participants with and without dietary supplements, most of the above significant results remained (Supplementary Table S8), except that the previous increased trends of serum 25(OH)D and folate concentrations turned not to be statistically significant among participants without dietary supplements (*p*-linear trend = 0.152 and 0.884, respectively). In addition, compared to participants without dietary supplements, higher concentrations of vitamin B6, B12, folate, 25(OH)D, total protein, HDL-C, and urinary iodine, and lower concentrations of blood lead, cadmium, mercury, and TC were observed among participants with dietary supplements (Supplementary Table S9).

## Discussion

Based on data from the nationally representative US surveys across 14 years (2003–2016), most nutritional biomarkers of US adults were improved, but some specific populations should be paid much attention to improve their nutritional status, especially for NHB and participants with low socioeconomic status. To our knowledge, this is the first study that reported the comprehensive evaluation of the trends of nutritional biomarkers and their status among US adults.

Trends of overall diet quality from 1999 to 2016 and dietary nutrients from 2003 to 2016 among US adults have been investigated by Rehm et al. and this study, respectively (24, 25). The analyzed data in these two studies were all from a 24-h dietary recall interview. Besides these, a total of 24 nutritional biomarkers were measured in the NHANES, which provided data on evaluating diet and nutritional status with less error and objective assessment. However, evidence on assessing the trends of all nutritional biomarkers measured in NHANES is limited. In the present study, most nutritional biomarker status was improved from 2003 to 2016, which adds further strength to the previous data that diet quality and dietary nutrients were improved among US adults (24, 25).

It is to be noted that although most nutritional biomarkers were improved among US adults, participants aged 40**–**59 years, men, NHB, and participants with low socioeconomic status should be paid more attention to due to their relatively poor status of nutritional biomarkers. These results were in line with previous reports for these specific populations (24, 25). The improved nutritional status could be achieved by giving different recommendations for a specific nutrient.

Iodine, cadmium, mercury, LDL-C, HDL-C, TG, and TC should be the main nutrients to be improved for participants aged 40–59. These adults should be encouraged to intake more seafood particularly rich in iodine and iodized salts (26, 27), fewer food crops, shellfish containing cadmium (28), and predatory fish with much methylmercury (29). In addition, they should intake more dietary fiber, red yeast rice, hawthorn fruit, garlic, and seaweed but less processed meat and cream, which could efficiently lower serum LDL-C, TG, and TG concentrations and increase the HDL-C concentration (30, 31). Furthermore, in order to decrease the LDL-C/HDL-C ratio among men, increasing serum HDL-C and decreasing LDL-C concentrations would be the main means, which could be attained by encouraging them to intake more fish and their products, nuts, and nutraceuticals containing phytosterols and isoflavones, and less fried food (32, 33).

Vitamin B_6_ and D, mercury, potassium, and iodine would be the key nutrients to be improved for NHB. More foods rich in vitamin B_6_ and vitamin D, such as eggs, chicken, fish, beans, and nuts, exposing adequate sunlight, fresh fruits, vegetables, which are generally good sources of potassium (34, 35), and seafood particularly rich in iodine (36), but less predatory fish with much methylmercury (29), should be recommended for them. Vitamin B_6_ and D, cadmium, lead and iodine, HDL-C, TG, and HDL-C/LDL-C ratio were all needed to be better for participants with low socioeconomic status. The suggestions stated above should be applied to them as well. Moreover, government and non-governmental organizations allocated funds for them and educated them on nutrition and health may be effective in improving their nutritional status (37, 38). In particular, this vulnerable population should be the focus of attention because of their relatively poor nutritional status.

Besides the above nutrients, serum concentrations of total calcium, proteins, and vitamin E may need to be paid attention to based on their decreased trends. For total calcium, the mean concentrations from 2003 to 2016 were 2.37 nmol/L for men, which was lower than the 2.81 nmol/L for Indian men and 2.42 nmol/L for men in the literature review (39, 40). In order to improve the nutritional status of calcium, men should be recommended to intake more dairy products involving milk, yogurt, and cheese, which are the best dietary source for calcium (41). Although the decreased trend was observed for total calcium for women, the mean concentration for it across 14 years was 2.35 nmol/L, which was comparable to the 2.31 nmol/L for women in the literature review (39, 42). In addition, the mean concentration of serum proteins was 71.11 g/L across 14 years, which was lower than the 74.80 g/L in the Korean cross-sectional study and 74.90 g/L in the Coronary Artery Risk Development in Adults (CARDIA, 1985–2011) study (43, 44). US adults should be encouraged to intake more seafood and plants, such as whole grains, legumes, and nuts, but fewer animal foods, such as unprocessed red meats and processed meats, to improve nutritional status for protein (24). Furthermore, the vitamin E concentration decreased from 29.39 to 27.37 μmol/L, but either of them was higher than the mean of 24.71 μmol/L in the NHANES (1999–2002) (45). Meanwhile, the prevalence of vitamin E deficiency was <1% in the present study, which was consistent with the result in the Second National Nutrition Report for US Population (46). Therefore, this slight decrease may not need to raise concerns.

In particular, dietary supplement products are widely used to maintain health and improve nutritional status in the US (47), and 52.3% of participants reported that they have eaten dietary supplements in the present study. As expected, nutritional status for all biomarkers was better among participants using dietary supplements than it among participants without supplements. However, some randomized controlled trials reported adverse outcomes for dietary supplements, in which additional nutrient intake from supplements may lead to intakes above the tolerable upper intake level (UL), especially for those nutrients that are fortified in foods (48). Although no concentrations above UL were observed in the present study, using dietary supplements should be cautious and it is better to follow medical guidance.

The present study has some merits to mention. The trends of nutritional biomarkers were measured from large nationally representative US surveys across 14 years. In addition, all biomarkers were measured by the CDC Nutrition Biomarker Laboratory, which has a well-established quality architecture based on laboratory policy and procedure manuals to ensure high-quality data. Of course, several limitations existed in the present study. First, differences in laboratory instruments, methods, and staff may influence the actual or measured concentrations of biomarkers, but the converted regression equations and equivalent data were used to match differences in laboratory methods in the NHANES survey years (3). Second, urinary concentrations of sodium and potassium are considered the gold standard measurements but they were not available and instead of serum concentrations in the NHANES. Third, the iron-status indicators were limited by only measured among women 12–49 years in the NHANES.

## Conclusion

In summary, most nutritional biomarkers were improved among US adults from 2003 to 2016. But further improvements of some nutrients are needed for the specific population especially for NHB and populations with low socioeconomic status, such as vitamin B_6_ and vitamin D, lead, cadmium, mercury, iodine, potassium, LDL-C, TG, TC, and HDL-C.

## Data Availability Statement

The datasets presented in this study can be found in online repositories. The names of the repository/repositories and accession number(s) can be found at: https://www.cdc.gov/nchs/nhanes/.

## Ethics Statement

The studies involving human participants were reviewed and approved by National Center for Health Statistics (NCHS) Research Ethics Review Board. The patients/participants provided their written informed consent to participate in this study.

## Author Contributions

XW contributed to the conceptualization and design of the study, supervised the data collection, statistical analyses, initial drafting of the manuscript, and reviewed and revised the manuscript. WW conceptualized and designed the study, completed the statistical analyses, drafted the initial manuscript, and reviewed and revised the manuscript. FZ, LW, and SH assisted with the data interpretation, reviewed, and revised the manuscript. All authors read and approved the final manuscript.

## Funding

This work was supported by grants from the National Natural Science Foundation of China (Nos. 82073536 and 81573134).

## Author Disclaimer

The content was solely the responsibility of the authors and does not necessarily represent the official views of the National Cancer Institute or the National Institutes of Health.

## Conflict of Interest

The authors declare that the research was conducted in the absence of any commercial or financial relationships that could be construed as a potential conflict of interest.

## Publisher's Note

All claims expressed in this article are solely those of the authors and do not necessarily represent those of their affiliated organizations, or those of the publisher, the editors and the reviewers. Any product that may be evaluated in this article, or claim that may be made by its manufacturer, is not guaranteed or endorsed by the publisher.
