# Supplementary material for: Trends in Nutritional Biomarkers by Demographic Characteristics Across 14 Years Among US Adults
Source: Front Nutr. 2022 Jan 13;8:737102. doi: 10.3389/fnut.2021.737102 (PMC8793029; doi:10.3389/fnut.2021.737102)
Supplement: Supplementary file 1 [file Data_Sheet_1.docx]

**Supplementary Table 1**

The sample size of each biomarker in National Health and Nutrition Examination Survey (2003-2016)

| Characteristics | 2003-  04 | 2005-06 | 2007-08 | 2009-10 | 2011-  12 | 2013-  14 | 2015-16 |
| --- | --- | --- | --- | --- | --- | --- | --- |
| Vitamin A | 4280 | 4164 | — | — | — | — | — |
| Vitamin B_6_ | 4279 | 4211 | 5277 | 5188 | — | — | — |
| Serum folate | 4311 | 4280 | 5301 | 5677 | 4828 | 5313 | 5116 |
| RBC folate | 4308 | 4717 | 5303 | 5700 | 4953 | 5294 | 5128 |
| Vitamin B_12_ | 4310 | 4120 | — | — | 4842 | 5271 | — |
| Vitamin C | 4263 | 4179 | — | — | — | — | — |
| 25-hydroxyvitamin D | 4318 | 4217 | 4568 | 5685 | 4927 | 5320 | — |
| Vitamin E | 4280 | 4164 | — | — | — | — | — |
| Ferritin | 1013 | 1074 | 1285 | 1537 | — | — | — |
| Iron | 1294 | 1378 | — | — | — | — | — |
| Potassium | 4279 | 4185 | 5272 | 5639 | 4852 | 5283 | 5104 |
| Sodium | 4279 | 4185 | 5272 | 5639 | 4852 | 5283 | 5104 |
| Phosphorus | 4279 | 4184 | 5274 | 5635 | 4854 | 5282 | 5103 |
| Total calcium | 4280 | 4185 | 5272 | 5639 | 4852 | 5254 | 5104 |
| Lead | 4345 | 4231 | 5323 | 5720 | 4995 | 2673 | 2595 |
| Mercury | 4345 | 4231 | 5323 | 5720 | 4995 | 2673 | 2595 |
| Cadmium | 4345 | 4231 | 5323 | 5720 | 4995 | 2673 | 2595 |
| Iodine | 1428 | 1474 | 5428 | 2015 | 1703 | 1798 | 1788 |
| Total protein | 4277 | 4175 | 5266 | 5627 | 4845 | 5274 | 5100 |
| Albumin | 4280 | 4185 | 5274 | 5639 | 4854 | 5283 | 5103 |
| Apolipoprotein B |  | 2008 | 2594 | 2739 | 2422 | 2537 | 2234 |
| HDL-C | 4300 | 4203 | 5291 | 5652 | 4878 | 5297 | 5111 |
| LDL-C | 1984 | 1946 | 2532 | 2691 | 2376 | 2498 | 2211 |
| Total Cholesterol | 4301 | 4203 | 5291 | 5652 | 4878 | 5297 | 5111 |
| Triglycerides | 2043 | 2000 | 2596 | 2739 | 2421 | 2538 | 2235 |

HDL-C, High-Density Lipoprotein Cholesterol; LDL-C, Low-Density Lipoprotein Cholesterol

**Supplementary Table 2**

Summary of nutritional biomarkers survey characteristics and laboratory assay methodology in National Health and Nutrition Examination Survey (2003-2016)

| Biomarker | Surveys available for trend analysis | Sample size | Laboratory assay method |
| --- | --- | --- | --- |
| Vitamin A | 2003-2006 | 8444 | Isocratic HPLC |
| Plasma vitamin B_6_ | 2003-2010 | 19255 | Reversed-phase HPLC with fluorometric detection |
| Serum and RBC folate | 2003-2016 | 8491(serum)  8525(RBC) | Quantaphase II Folate radioassay kit (2003-2006)  microbiological assay (2007-2016) |
| Vitamin B_12_ | 2003-2006, 2011-2014 | 18543 | Quantaphase II Vitamin B12 radioassay kit (2003-2006)  Roche electrochemiluminescence immunoassay (2011-2014) |
| Vitamin C | 2003-2006 | 8442 | Isocratic HPLC |
| 25-hydroxyvitamin D | 2003-2014 | 29035 | Diasorin RIA (1988–2006); (LC-MS/MS) (2007–2014) |
| Vitamin E | 2003-2006 | 8444 | Isocratic HPLC |
| Ferritin | 2003-2010 | 4909 | Immuno-turbidimetry |
| Iron | 2003-2006 | 2672 | Timed-endpoint |
| Potassium | 2003-2016 | 34611 | LX system utilizes indirect I.S.E. methodology (2003-2012); DxC800 system (2013-2016) |
| Sodium | 2003-2016 | 34614 | LX system utilizes indirect I.S.E. methodology (2003-2012); DxC800 system (2013-2016) |
| Phosphorus | 2003-2016 | 34611 | LX system uses a timed-rate method(2003-2012)  DxC800 system(2013-2016) |
| Total calcium | 2003-2016 | 34586 | DxC800 system uses indirect I.S.E. methodology (2003-2012); DxC800 system(2013-2016) |
| urinary iodine | 2003-2016 | 15454 | ICP-DRC-MS(2003-2016) |
| Total protein | 2003-2016 | 34564 | DxC800 uses a timed rate biuret method (2003-2016) |
| Apolipoprotein B | 2005-2016 | 14534 | Dade-Behring BN100 nepholometric immunoassay (2005-2006); Dade-Behring BN ProSpec nepholometric immunoassay (2007-2016) |
| HDL-C | 2003-2016 | 29621 | direct immunoassay method (2003-2016) |
| Total Cholesterol | 2003-2016 | 14337 | Enzymatic assay (2003-2016) |
| Triglycerides | 2003-2016 | 29622 | Enzymatic assay (2003-2016) |

Nutrients were measured in serum unless otherwise stated. HDL-C, High-Density Lipoprotein Cholesterol;

**Supplementary Table 3**

The quality control coefficient of variation (CV) of each biomarker in National Health and Nutrition Examination Survey (2003-2016)

| Characteristics | 2003-04 | 2005-  06 | 2007-  08 | 2009-  10 | 2011-  12 | 2013-  14 | 2015-  16 |
| --- | --- | --- | --- | --- | --- | --- | --- |
| Vitamin A | 2.6-2.9 | 3.1-3.5 | — | — | — | — | — |
| Vitamin B_6_ | 2.8-9.6 | 2.5-8.3 | 1.7-6.7 | 3.5-7.1 | — | — | — |
| Serum folate | 3.2-6.3 | 2.5-8.7 | 6.9-8.9 | 4.7-8.5 | 5.3-12.3 | 4.8-11.3 | 3.1-8.5 |
| RBC folate | 1.7-4.3 | 1.6-3.6 | 6.7-12.1 | 7.5-8.2 | 5.4-8.0 | 6.7-7.1 | 4.6-6.7 |
| Vitamin B_12_ | 2.1-4.1 | 1.7-4.4 | — | — | 2.4-2.9 | 0.8-3.6 | — |
| Vitamin C | 1.7-7.6 | 3.7-5.7 | — | — | — | — | — |
| 25-hydroxyvitamin D | 4.4-11.4 | 4.4-11.4 | 4.6-11.5 | 4.5-9.0 | 3.3-8.7 | 3.5-8.4 | — |
| Vitamin E | 2.5-5.2 | 2.4-2.5 | — | — | — | — | — |
| Ferritin | 3.0-8.0 | 4.3-5.4 | 1.9-10.6 | 2.9-3.8 | — | — | — |
| Iron | 1.5-2.1 | 1.2-2.2 | — | — | — | — | — |
| Potassium | 0.9-1.6 | 0.9-1.6 | 0.7-4.3 | 0.9-2.2 | 0.8-2.1 | 0.7-1.1 | 0.8-1.2 |
| Sodium | 0.8-1.0 | 0.7-1.0 | 0.6-1.1 | 0.7-1.1 | 0.7-1.0 | 0.5-1.0 | 0.6-1.3 |
| Phosphorus | 1.1-1.6 | 1.3-1.6 | 1.8-2.1 | 1.0-1.6 | 0.9-1.9 | 1.0-1.5 | 1.1-1.8 |
| Total calcium | 0.9-1.2 | 1.0-1.3 | 0.8-2.8 | 0.9-2.8 | 0.9-1.2 | 0.7-1.3 | 0.6-1.4 |
| Lead | 1.7-4.4 | 1.4-2.1 | 1.6-2.3 | 1.2-1.7 | 2.4-3.8 | 1.0-3.0 | 1.5-3.1 |
| Mercury | 2.7-8.1 | 2.6-21.4 | 2.4-16.3 | 2.3-15.1 | 3.9-10.9 | 2.4-10.7 | 2.9-10.2 |
| Cadmium | 3.2-9.4 | 3.4-10.9 | 3.4-11.3 | 2.9-10.3 | 3.1-13.7 | 2.4-8.3 | 2.6-8.8 |
| Arsenic | 2.6-9.2 | 3.7-8.4 | 3.3-8.7 | 2.9-6.0 | 1.9-8.3 | 0.7-2.2 | 3.9-6.1 |
| Iodine | 1.4-2.4 | 1.0-1.9 | 2.0-2.5 | 1.6-2.5 | 1.6-2.6 | 4.1-6.0 | 4.4-4.7 |
| Total protein | 2.1-2.3 | 1.1-1.7 | 1.1-1.7 | 1.2-1.6 | 1.4-1.8 | 1.4-2.2 | 1.4-1.5 |
| Albumin | 1.5-1.8 | 0.7-1.5 | 0.9-1.2 | 0.8-1.1 | 0.8-1.0 | 0.7-1.0 | 0.9-1.0 |
| Apolipoprotein B |  | 1.1-2.9 | 2.0-6.2 | 2.9-4.6 | 2.4-5.8 | 0.7-7.4 | 2.0-2.7 |
| HDL-C | 1.7-2.3 | 1.9-2.6 | 1.5-3.2 | 2.0-3.5 | 2.2-3.7 | 1.7-4.6 | 1.6-2.5 |
| Total Cholesterol | 1.2-1.4 | 1.1-1.3 | 1.2-1.6 | 1.1-1.3 | 1.3-1.5 | 1.1-1.6 | 1.2-1.3 |
| Triglycerides | 1.5-2.1 | 1.8-2.1 | 1.3-2.4 | 1.4-2.4 | 2.0-3.0 | 1.5-2.2 | 1.6-2.1 |

HDL-C, High-Density Lipoprotein Cholesterol

**Supplementary Table 4**

The mean (SE) of different types of dietary supplement use among U.S adults in the National Health and Nutrition Examination Survey (2003-2016)

|  | All population | Men | Women |
| --- | --- | --- | --- |
| Vitamins |  |  |  |
| Thiamin (mg) | 3.60(0.28) | 3.42(0.42) | 3.76(0.31) |
| Riboflavin (mg) | 2.28(0.13) | 2.19(0.18) | 2.36(0.14) |
| Niacin (mg) | 10.43(0.57) | 11.17(0.97) | 9.73(0.49) |
| Vitamin B_6_ (mg) | 3.41(0.37) | 2.65(0.16) | 4.12(0.70) |
| Folate, DFE (mcg) | 215.17(4.96) | 181.56(6.46) | 246.85(5.96) |
| Vitamin B12 (mcg) | 57.77(3.72) | 41.36(3.01) | 73.22(6.27) |
| Vitamin C (mg) | 84.97(3.07) | 80.52(3.63) | 89.15(4.05) |
| Vitamin E (mg) |  |  |  |
| Vitamin D (mcg) | 10.70(0.49) | 7.48(0.34) | 13.73(0.79) |
| Minerals |  |  |  |
| Calcium (mg) | 155.61(3.58) | 92.08(2.86) | 215.44(5.34) |
| Phosphorus (mg) | 8.63(0.38) | 8.46(0.56) | 8.80(0.43) |
| Magnesium (mg) | 29.95(1.11) | 26.46(1.00) | 33.24(1.54) |
| Iron (mg) | 3.14(0.08) | 1.76(0.09) | 4.45(0.14) |
| Zinc (mg) | 4.21(0.12) | 3.92(0.21) | 4.47(0.10) |
| Copper (mg) | 0.19(0.01) | 0.17(0.01) | 0.20(0.01) |
| Selenium (mcg) | 16.23(1.48) | 17.36(0.58) | 15.16(2.90) |
| Potassium (mg) | 14.93(0.48) | 17.00(0.65) | 12.98(0.57) |
| Sodium (mg) | 2.31(0.18) | 2.66(0.27) | 1.98(0.18) |
| Protein |  |  |  |
| Protein (gm) | 0.09(0.01) | 0.15(0.03) | 0.05(0.01) |
| Lipids |  |  |  |
| Total saturated fatty acids (gm) | 0.02(0.00) | 0.03(0.00) | 0.02(0.00) |
| Total monounsaturated fatty acids (gm) | 0.01(0.00) | 0.01(0.00) | 0.01(0.00) |
| Total polyunsaturated fatty acids (gm) | 0.04(0.00) | 0.04(0.00) | 0.05(0.00) |

**Supplementary Table 5**

Summary of information on reference values or cutoffs points for nutritional biomarkers in the National Health and Nutrition Examination Survey, 2003-2016

| Nutritional Biomarkers | Recommended biochemical concentrations (reference) |
| --- | --- |
| Vitamin A | Deficiency: vitamin A < 0.7 umol/L |
| Vitamin B_6_ | Deficiency: PLP < 20 nmol/L; insufficiency: PLP 20-30 nmol/L |
| Serum and RBC folate | Deficiency: serum folate < 3ng/mL; insufficiency: serum folate 3-6 ng/mL  Deficiency: RBC folate < 140 ng/mL; insufficiency: RBC folate 140-160 ng/mL |
| Vitamin B_12_ | Deficiency: vitamin B12 < 200 ng/mL |
| Vitamin C | Deficiency: vitamin C < 11.4 umol/L |
| 25-hydroxyvitamin D | Deficiency: 25(OH)D < 30 nmol/L; insufficiency: 25(OH)D 30-50 nmol/L  Reason for concern: 25(OH)D > 125 nmol/L |
| Vitamin E | Deficiency: a-Tocopherol < 11.6 mmol/L |
| Iron | Deficiency: serum Ferritin <15 ug/L |
| Iodine | Deficiency: urinary iodine < 100 ng/mL; Excessive intake: urinary iodine > 200 ng/mL |
| Phosphorus | Hypophosphatemia: serum phosphorus < 0.80 mmol/L |
| Potassium | Hypokalemia: serum Potassium < 3.5 mmol/L; Hyperkalemia: serum potassium > 5.0 mmol/L |
| Sodium | Hyponatremia: serum sodium < 135 mmol/L; Hypernatremia: serum sodium > 145 mmol/L |
| Total calcium | Hypocalcemia: serum calcium < 2.0 mmol/L |
| Lead | Elevated blood lead: blood lead ≥ 5µg/dL |
| Cadmium | Elevated blood cadmium: blood cadmium ≥ 1.0µg/L |
| Mercury | Elevated blood mercury: blood mercury ≥ 3.5µg/L; blood mercury ≥ 5.8µg/L |
| Albumin  Protein | Hypoalbuminemia: serum albumin < 35g/L  Hypoproteinemia: serum total protein < 60g/L |
| Dyslipidemia | Elevated Apo B: apolipoprotein B >130 mg/dL |
|  | Lowered HDL-C: HDL-C <40 mg/dL in men and <50 mg/dL in women |
|  | Elevated LDL-C: LDL-C >130 mg/dL |
|  | Lowered HDL/LDL: HDL/LDL ratio <0.4 |
|  | Elevated TC: total cholesterol >200 mg/dL |
|  | Elevated TG: triglycerides >150 mg/dL |

Abbreviation: PLP, pyridoxal-5’-phosphate; 25(OH) D, 25-hydroxyvitamin D; Apo B, apolipoprotein B; HDL-C, High-Density Lipoprotein Cholesterol; LDL-C, Low-Density Lipoprotein Cholesterol; TC, Total Cholesterol; TG, Triglyceride

**Supplementary Table 6**

Trends in mean (SE) or geometric mean (SE) of nutritional biomarkers by demographic variables in National Health and Nutrition Examination Survey, 2003-2016^1^

| Characteristics | 2003-2004 | 2005-2006 | 2007-2008 | 2009-2010 | 2011-2012 | 2013-2014 | 2015-2016 | *P*-linear^2^ | *P-*quadratic |
| --- | --- | --- | --- | --- | --- | --- | --- | --- | --- |
|  | **Vitamins** | | | | | | |  |  |
| **Vitamin B_6_ (nmol/L)^1^** |  |  |  |  |  |  |  |  |  |
| Age group, y |  |  |  |  |  |  |  |  |  |
| 20–39 y | 38.38(2.50) | 51.50(1.85) | 51.20(2.16) | 50.50(2.40) | — | — | — | 0.001 | 0.001 |
| 40–59 y | 44.60(2.44) | 49.01(2.17) | 50.44(2.98) | 48.27(1.14) | — | — | — | 0.132 | 0.005 |
| ≥60 y | 52.52(3.28) | 50.44(2.07) | 54.71(2.52) | 47.45(1.42) | — | — | — | 0.229 | 0.189 |
| Sex |  |  |  |  |  |  |  |  |  |
| Male | 54.64(2.70) | 54.87(1.62) | 55.11(2.11) | 53.16(1.53) | — | — | — | 0.652 | 0.569 |
| Female^3^ | 35.46(1.89) | 46.13(1.49) | 48.67(1.80) | 45.10(1.07) | — | — | — | <0.001 | <0.001 |
| Race-ethnicity |  |  |  |  |  |  |  |  |  |
| NHW | 46.32(2.86) | 52.41(1.82) | 54.18(2.89) | 51.34(1.61) | — | — | — | 0.131 | 0.017 |
| NHB | 33.23(1.64) | 38.71(1.79) | 40.66(1.92) | 36.83(0.77) | — | — | — | 0.045 | 0.003 |
| MA | 38.65(2.83) | 47.87 (1.42) | 47.31(2.15) | 47.01(1.18) | — | — | — | 0.026 | 0.027 |
| Socioeconomic status |  |  |  |  |  |  |  |  |  |
| Low | 32.20(2.12) | 35.75(2.24) | 36.87(2.43) | 33.29(2.60) | — | — | — | 0.655 | 0.037 |
| Medium^3^ | 39.54(2.72) | 45.75(1.27) | 48.64(2.19) | 44.44(1.87) | — | — | — | 0.032 | 0.003 |
| High | 53.88(2.09) | 59.41(1.53) | 59.72(1.65) | 59.69(1.26) | — | — | — | 0.089 | 0.199 |
| **RBC folate(ng/mL)^1^** |  |  |  |  |  |  |  |  |  |
| Age group, y |  |  |  |  |  |  |  |  |  |
| 20–39 y | 418.89(8.32) | 450.82(8.58) | 443.71(9.09) | 411.62(5.59) | 421.70(9.68) | 455.41(7.89) | 456.35(8.25) | 0.096 | 0.073 |
| 40–59 y | 468.27(16.44) | 492.04(9.84) | 494.77(16.62) | 472.61(10.61) | 467.09(10.27) | 511.45(10.23) | 506.41(9.87) | 0.138 | 0.356 |
| ≥60 y | 560.14(15.13) | 596.48(15.01) | 620.01(15.68) | 560.14(7.20) | 557.31(13.79) | 595.94(11.22) | 581.30(12.34) | 0.138 | 0.822 |
| Sex |  |  |  |  |  |  |  |  |  |
| Male | 452.58(11.66) | 487.42(8.00) | 480.62(8.92) | 456.16(8.53) | 457.72(8.67) | 501.19(9.87) | 498.88(10.02) | 0.033 | 0.260 |
| Female | 482.04(12.71) | 504.76(8.84) | 516.34(11.88) | 481.36(6.50) | 484.73(7.96) | 520.95(12.34) | 516.18(11.78) | 0.469 | 0.421 |
| Race-ethnicity |  |  |  |  |  |  |  |  |  |
| NHW | 492.66(17.92) | 523.38(7.35) | 535.54(16.46) | 505.68(8.88) | 504.78(8.79) | 549.29(10.26) | 544.50(10.23) | 0.027 | 0.748 |
| NHB | 378.62(7.24) | 401.16(6.19) | 403.84(9.18) | 381.00(4.98) | 381.42(7.25) | 398.47(7.37) | 419.47(6.46) | <0.001 | 0.050 |
| MA | 422.35(13.21) | 449.49(6.99) | 435.44(18.21) | 403.56(9.25) | 434.81(9.25) | 466.02(8.89) | 455.93(8.78) | 0.084 | 0.064 |
| Socioeconomic status |  |  |  |  |  |  |  |  |  |
| Low | 428.05(13.54) | 434.91(10.15) | 440.86(12.84) | 408.98(8.17) | 434.61(8.98) | 450.71(8.72) | 453.63(9.67) | 0.111 | 0.147 |
| Medium | 468.49(11.22) | 488.65(8.09) | 496.82(12.67) | 465.69(9.72) | 462.06(11.24) | 507.81(10.25) | 498.42(10.97) | 0.041 | 0.194 |
| High | 476.21(17.96) | 519.64(10.14) | 527.23(11.88) | 498.20(6.28) | 492.83(9.63) | 535.30(12.67) | 530.27(11.98) | 0.060 | 0.971 |
| **Vitamin B_12_ (pg/mL)^1^** |  |  |  |  |  |  |  |  |  |
| Age group, y |  |  |  |  |  |  |  |  |  |
| 20–39 y | 453.31(9.16) | 463.55(7.81) | — | — | 516.54(5.14) | 502.69(5.19) | — | <0.001 | 0.069 |
| 40–59 y | 459.94(13.28) | 472.06(10.66) | — | — | 511.21(8.61) | 524.56(8.90) | — | <0.001 | 0.992 |
| ≥60 y | 476.98(9.71) | 486.74(11.05) | — | — | 578.36(14.19) | 556.03(13.99) | — | <0.001 | 0.108 |
| Sex |  |  |  |  |  |  |  |  |  |
| Male | 459.09(7.27) | 464.94(7.31) | — | — | 521.43(9.58) | 505.94(6.49) | — | <0.001 | 0.132 |
| Female | 463.34(11.21) | 478.74(10.35) | — | — | 537.65(6.20) | 543.38(5.32) | — | <0.001 | 0.479 |
| Race-ethnicity |  |  |  |  |  |  |  |  |  |
| NHW | 449.22(9.94) | 461.92(9.28) | — | — | 520.70(8.87) | 516.37(6.25) | — | <0.001 | 0.289 |
| NHB | 518.32(9.68) | 516.45(9.83) | — | — | 575.02(11.40) | 552.78(9.37) | — | <0.001 | 0.276 |
| MA | 510.37(15.80) | 499.03(14.12) | — | — | 550.55(8.97) | 547.44(20.25) | — | 0.013 | 0.840 |
| Socioeconomic status |  |  |  |  |  |  |  |  |  |
| Low | 485.06(11.99) | 466.34(7.49) | — | — | 499.46(9.20) | 522.88(9.64) | — | 0.015 | 0.140 |
| Medium | 459.09(11.52) | 470.11(8.56) | — | — | 524.81(9.76) | 523.36(8.34) | — | <0.001 | 0.432 |
| High | 450.14(6.55) | 473.91(13.63) | — | — | 536.29(7.50) | 528.08(8.21) | — | <0.001 | 0.172 |
| **25(OH)D (nmol/L)** |  |  |  |  |  |  |  |  |  |
| Age group, y |  |  |  |  |  |  |  |  |  |
| 20–39 y | 62.58(1.88) | 61.97(1.44) | 65.88(1.68) | 62.97(1.64) | 62.49(1.49) | 61.11(1.53) | — | 0.57 | 0.200 |
| 40–59 y | 62.15(1.98) | 60.13(1.16) | 67.03(1.33) | 68.67(1.33) | 70.88(2.19) | 68.88(1.46) | — | <0.001 | 0.208 |
| ≥60 y | 62.50(1.17) | 59.45(1.14) | 68.98(1.07) | 72.62(1.59) | 81.70(1.73) | 81.36(1.37) | — | <0.001 | 0.525 |
| Sex |  |  |  |  |  |  |  |  |  |
| Male | 62.92(1.77) | 60.71(0.95) | 65.46(1.22) | 65.62(1.33) | 67.67(1.62) | 65.82(1.16) | — | 0.004 | 0.482 |
| Female | 61.89(1.58) | 60.57(1.32) | 68.64(0.94) | 69.45(1.60) | 73.54(1.81) | 72.95(1.66) | — | <0.001 | 0.388 |
| Race-ethnicity |  |  |  |  |  |  |  |  |  |
| NHW | 67.76(1.68) | 65.40(0.99) | 73.71(0.88) | 74.67(1.22) | 77.87(1.35) | 75.91(1.35) | — | <0.001 | 0.766 |
| NHB | 40.57(1.41) | 41.73(1.10) | 42.08(1.44) | 46.58(2.16) | 50.71(1.24) | 50.23(1.42) | — | <0.001 | 0.159 |
| MA | 53.55(1.76) | 50.89(1.86) | 53.71(1.76) | 53.93(0.70) | 54.27(1.82) | 55.39(2.12) | — | 0.022 | 0.587 |
| Socioeconomic status |  |  |  |  |  |  |  |  |  |
| Low | 55.99(3.38) | 52.91(1.72) | 58.16(2.74) | 57.61(1.64) | 63.02(3.39) | 60.61(1.83) | — | <0.001 | 0.901 |
| Medium | 61.08(1.62) | 59.03(1.20) | 65.16(1.25) | 65.79(1.78) | 67.03(1.39) | 66.53(1.51) | — | <0.001 | 0.442 |
| High | 65.80(1.84) | 63.95(1.17) | 71.38(0.87) | 72.49(1.18) | 77.06(2.11) | 75.20(1.45) | — | <0.001 | 0.337 |
| **Vitamin E (umol/L)^1^** |  |  |  |  |  |  |  |  |  |
| Age group, y |  |  |  |  |  |  |  |  |  |
| 20–39 y | 24.09(0.65) | 23.56(0.23) | — | — | — | — | — | 0.08 | — |
| 40–59 y | 30.78(0.65) | 28.52(0.42) | — | — | — | — | — | 0.003 | — |
| ≥60 y | 37.65(0.76) | 32.47(0.53) | — | — | — | — | — | <0.001 | — |
| Sex |  |  |  |  |  |  |  |  |  |
| Male | 28.79(0.55) | 26.80(0.27) | — | — | — | — | — | 0.001 | — |
| Female | 30.00(0.48) | 27.93(0.39) | — | — | — | — | — | 0.001 | — |
| Race-ethnicity |  |  |  |  |  |  |  |  |  |
| NHW | 30.75(0.70) | 28.36(0.32) | — | — | — | — | — | 0.002 | — |
| NHB | 24.70(0.31) | 23.18(0.21) | — | — | — | — | — | <0.001 | — |
| MA | 25.93(0.49) | 25.81(0.39) | — | — | — | — | — | 0.85 | — |
| Socioeconomic status |  |  |  |  |  |  |  |  |  |
| Low | 27.06(0.32) | 24.90(0.33) | — | — | — | — | — | 0.005 | — |
| Medium | 28.48(0.47) | 26.47(0.42) | — | — | — | — | — | 0.001 | — |
| High | 31.32(0.37) | 28.98(0.35) | — | — | — | — | — | 0.004 | — |
|  | **Minerals** | | | | | | |  |  |
| **Phosphorus (mmol/L)** |  |  |  |  |  |  |  |  |  |
| Age group, y |  |  |  |  |  |  |  |  |  |
| 20–39 y | 1.24(0.007) | 1.25(0.004) | 1.22(0.004) | 1.22(0.004) | 1.22(0.005) | 1.24(0.005) | 1.21(0.008) | 0.002 | 0.564 |
| 40–59 y | 1.22(0.007) | 1.22(0.005) | 1.21(0.007) | 1.20(0.006) | 1.20(0.007) | 1.23(0.005) | 1.18(0.01) | 0.03 | 0.334 |
| ≥60 y | 1.21(0.004) | 1.23(0.006) | 1.22(0.006) | 1.21(0.003) | 1.19(0.006) | 1.23(0.009) | 1.19(0.01) | 0.01 | 0.260 |
| Sex |  |  |  |  |  |  |  |  |  |
| Male | 1.21(0.006) | 1.21(0.005) | 1.19(0.005) | 1.18(0.004) | 1.18(0.003) | 1.21(0.005) | 1.17(0.008) | <0.001 | 0.885 |
| Female | 1.24(0.005) | 1.25(0.004) | 1.24(0.005) | 1.24(0.003) | 1.23(0.005) | 1.26(0.006) | 1.22(0.008) | 0.02 | 0.301 |
| Race-ethnicity |  |  |  |  |  |  |  |  |  |
| NHW | 1.23(0.006) | 1.24(0.004) | 1.22(0.006) | 1.22(0.004) | 1.21(0.003) | 1.24(0.007) | 1.19(0.01) | 0.007 | 0.407 |
| NHB | 1.23(0.007) | 1.21(0.005) | 1.21(0.007) | 1.20(0.009) | 1.20(0.007) | 1.22(0.007) | 1.18(0.01) | 0.01 | 0.873 |
| MA | 1.23(0.008) | 1.23(0.006) | 1.20(0.006) | 1.19(0.007) | 1.19(0.009) | 1.22(0.008) | 1.19(0.008) | 0.005 | 0.040 |
| Socioeconomic status |  |  |  |  |  |  |  |  |  |
| Low | 1.23(0.006) | 1.24(0.01) | 1.21(0.004) | 1.20(0.008) | 1.20(0.005) | 1.23(0.007) | 1.18(0.007) | 0.004 | 0.977 |
| Medium | 1.23(0.007) | 1.24(0.004) | 1.21(0.005) | 1.21(0.005) | 1.20(0.007) | 1.24(0.005) | 1.20(0.01) | 0.006 | 0.346 |
| High | 1.22(0.006) | 1.23(0.005) | 1.22(0.007) | 1.22(0.004) | 1.21(0.005) | 1.23(0.008) | 1.19(0.01) | 0.009 | 0.101 |
| **Total calcium (mmo/L)** |  |  |  |  |  |  |  |  |  |
| Age group, y |  |  |  |  |  |  |  |  |  |
| 20–39 y | 2.40(0.004) | 2.38(0.003) | 2.36(0.007) | 2.36(0.003) | 2.35(0.004) | 2.37(0.004) | 2.34(0.003) | <0.001 | 0.004 |
| 40–59 y | 2.38(0.004) | 2.37(0.004) | 2.35(0.007) | 2.36(0.004) | 2.34(0.004) | 2.35(0.004) | 2.33(0.004) | <0.001 | 0.111 |
| ≥60 y | 2.39(0.004) | 2.37(0.005) | 2.36(0.009) | 2.37(0.003) | 2.35(0.005) | 2.37(0.003) | 2.35(0.003) | <0.001 | 0.010 |
| Sex |  |  |  |  |  |  |  |  |  |
| Male | 2.40(0.003) | 2.38(0.004) | 2.36(0.007) | 2.37(0.004) | 2.35(0.004) | 2.37(0.003) | 2.35(0.004) | <0.001 | 0.004 |
| Female | 2.38(0.004) | 2.37(0.005) | 2.34(0.008) | 2.36(0.003) | 2.34(0.004) | 2.36(0.004) | 2.33(0.002) | <0.001 | 0.032 |
| Race-ethnicity |  |  |  |  |  |  |  |  |  |
| NHW | 2.39(0.004) | 2.38(0.004) | 2.35(0.009) | 2.37(0.003) | 2.35(0.004) | 2.37(0.004) | 2.35(0.003) | <0.001 | 0.004 |
| NHB | 2.39(0.005) | 2.38(0.005) | 2.36(0.004) | 2.37(0.008) | 2.35(0.004) | 2.36(0.004) | 2.34(0.005) | <0.001 | 0.611 |
| MA | 2.37(0.006) | 2.36(0.006) | 2.35(0.005) | 2.33(0.005) | 2.33(0.006) | 2.35(0.003) | 2.32(0.008) | <0.001 | 0.242 |
| Socioeconomic status |  |  |  |  |  |  |  |  |  |
| Low | 2.38(0.004) | 2.37(0.004) | 2.35(0.007) | 2.35(0.006) | 2.34(0.006) | 2.36(0.005) | 2.33(0.003) | <0.001 | 0.140 |
| Medium | 2.39(0.003) | 2.38(0.003) | 2.35(0.007) | 2.36(0.004) | 2.34(0.003) | 2.36(0.003) | 2.34(0.003) | <0.001 | 0.042 |
| High | 2.39(0.005) | 2.37(0.006) | 2.35(0.008) | 2.37(0.004) | 2.35(0.002) | 2.37(0.004) | 2.34(0.003) | <0.001 | 0.004 |
| **Lead (ug/dL)^1^** |  |  |  |  |  |  |  |  |  |
| Age group, y |  |  |  |  |  |  |  |  |  |
| 20–39 y | 1.20(0.02) | 1.04(0.02) | 1.05(0.03) | 0.91(0.02) | 0.76(0.02) | 0.70(0.01) | 0.66(0.01) | <0.001 | 0.411 |
| 40–49 y | 1.65(0.04) | 1.53(0.05) | 1.50(0.03) | 1.35(0.03) | 1.23(0.02) | 1.03(0.02) | 0.99(0.01) | <0.001 | 0.028 |
| ≥60 y | 2.04(0.07) | 2.07(0.06) | 1.84(0.05) | 1.67(0.03) | 1.55(0.03) | 1.38(0.03) | 1.31(0.03) | <0.001 | 0.263 |
| Sex |  |  |  |  |  |  |  |  |  |
| Male | 1.85(0.04) | 1.71(0.05) | 1.64(0.04) | 1.47(0.03) | 1.31(0.04) | 1.13(0.03) | 1.04(0.02) | <0.001 | 0.012 |
| Female | 1.29(0.03) | 1.20(0.03) | 1.18(0.02) | 1.05(0.02) | 0.94(0.02) | 0.85(0.01) | 0.82(0.01) | <0.001 | 0.302 |
| Race-ethnicity |  |  |  |  |  |  |  |  |  |
| NHW | 1.49(0.04) | 1.42(0.03) | 1.35(0.03) | 1.21(0.02) | 1.11(0.02) | 0.99(0.02) | 0.91(0.02) | <0.001 | 0.088 |
| NHB | 1.74(0.04) | 1.48(0.03) | 1.48(0.03) | 1.34(0.02) | 1.11(0.02) | 1.00(0.03) | 0.96(0.01) | <0.001 | 0.732 |
| MA | 1.68(0.04) | 1.48(0.03) | 1.41(0.03) | 1.38(0.03) | 0.99(0.01) | 0.88(0.01) | 0.87(0.01) | <0.001 | 0.213 |
| Socioeconomic status |  |  |  |  |  |  |  |  |  |
| Low | 1.98(0.04) | 1.78(0.04) | 1.63(0.03) | 1.56(0.04) | 1.35(0.03) | 1.09(0.05) | 1.12(0.04) | <0.001 | 0.549 |
| Medium | 1.54(0.03) | 1.49(0.02) | 1.38(0.04) | 1.21(0.05) | 1.07(0.03) | 0.95(0.04) | 0.91(0.03) | <0.001 | 0.330 |
| High | 1.44(0.03) | 1.29(0.03) | 1.32(0.02) | 1.18(0.04) | 1.07(0.03) | 0.99(0.03) | 0.87(0.03) | <0.001 | 0.083 |
| **Cadmium (ug/L)^1^** |  |  |  |  |  |  |  |  |  |
| Age group, y |  |  |  |  |  |  |  |  |  |
| 20–39 y | 0.32(0.01) | 0.32(0.02) | 0.33(0.01) | 0.31(0.01) | 0.28(0.01) | 0.26(0.01) | 0.24(0.01) | <0.001 | 0.002 |
| 40–49 y | 0.40(0.02) | 0.39(0.02) | 0.41(0.02) | 0.37(0.01) | 0.36(0.01) | 0.32(0.01) | 0.31(0.01) | <0.001 | 0.021 |
| ≥60 y | 0.48(0.02) | 0.45(0.02) | 0.41(0.01) | 0.42(0.02) | 0.40(0.01) | 0.35(0.01) | 0.35(0.01) | <0.001 | 0.569 |
| Sex |  |  |  |  |  |  |  |  |  |
| Male | 0.35(0.02) | 0.35(0.02) | 0.35(0.02) | 0.32(0.01) | 0.31(0.01) | 0.25(0.01) | 0.26(0.01) | <0.001 | 0.005 |
| Female | 0.41(0.02) | 0.40(0.02) | 0.40(0.02) | 0.39(0.01) | 0.37(0.02) | 0.34(0.02) | 0.33(0.01) | <0.001 | 0.015 |
| Race-ethnicity |  |  |  |  |  |  |  |  |  |
| NHW | 0.38(0.03) | 0.38(0.02) | 0.37(0.03) | 0.35(0.02) | 0.33(0.02) | 0.29(0.01) | 0.28(0.02) | <0.001 | 0.005 |
| NHB | 0.41(0.03) | 0.38(0.02) | 0.44(0.03) | 0.42(0.03) | 0.38(0.02) | 0.35(0.02) | 0.36(0.02) | 0.017 | 0.059 |
| MA | 0.31(0.02) | 0.30(0.02) | 0.30(0.01) | 0.30(0.02) | 0.28(0.01) | 0.24(0.01) | 0.26(0.01) | <0.001 | 0.546 |
| Socioeconomic status |  |  |  |  |  |  |  |  |  |
| Low | 0.52(0.03) | 0.43(0.02) | 0.52(0.02) | 0.49(0.01) | 0.46(0.01) | 0.38(0.01) | 0.44(0.01) | 0.045 | 0.466 |
| Medium | 0.41(0.03) | 0.42(0.02) | 0.39(0.01) | 0.38(0.01) | 0.36(0.01) | 0.31(0.01) | 0.31(0.01) | <0.001 | 0.044 |
| High | 0.32(0.01) | 0.32(0.01) | 0.33(0.02) | 0.31(0.01) | 0.29(0.01) | 0.25(0.01) | 0.25(0.01) | <0.001 | 0.001 |
| **Mercury (ug/L)^1^** |  |  |  |  |  |  |  |  |  |
| Age group, y |  |  |  |  |  |  |  |  |  |
| 20–39 y | 0.90(0.06) | 0.91(0.07) | 0.85(0.07) | 0.87(0.06) | 0.70(0.05) | 0.66(0.06) | 0.70(0.06) | <0.001 | 0.561 |
| 40–49 y | 1.08(0.07) | 1.20(0.08) | 1.07(0.06) | 1.16(0.08) | 0.94(0.06) | 0.91(0.05) | 0.90(0.04) | 0.001 | 0.102 |
| ≥60 y | 0.97(0.06) | 1.09(0.07) | 0.91(0.05) | 1.16(0.08) | 1.00(0.07) | 0.92(0.06) | 0.86(0.05) | 0.263 | 0.079 |
| Sex |  |  |  |  |  |  |  |  |  |
| Male | 1.03(0.07) | 1.08(0.07) | 1.02(0.06) | 1.09(0.06) | 0.90(0.04) | 0.82(0.06) | 0.82(0.06) | <0.001 | 0.048 |
| Female | 0.94(0.06) | 1.05(0.07) | 0.88(0.05) | 1.00(0.05) | 0.83(0.07) | 0.81(0.06) | 0.81(0.06) | 0.002 | 0.294 |
| Race-ethnicity |  |  |  |  |  |  |  |  |  |
| NHW | 0.94(0.07) | 1.08(0.09) | 0.89(0.07) | 1.12(0.09) | 0.83(0.07) | 0.78(0.05) | 0.74(0.05) | 0.001 | 0.122 |
| NHB | 1.16(0.11) | 1.03(0.10) | 0.97(0.08) | 1.16(0.09) | 0.85(0.08) | 0.86(0.06) | 0.85(0.05) | 0.001 | 0.866 |
| MA | 0.68(0.06) | 0.71(0.07) | 0.73(0.07) | 0.76(0.08) | 0.61(0.05) | 0.59(0.05) | 0.65(0.06) | 0.134 | 0.279 |
| Socioeconomic status |  |  |  |  |  |  |  |  |  |
| Low | 0.66(0.03) | 0.70(0.04) | 0.65(0.03) | 0.75(0.04) | 0.61(0.03) | 0.57(0.03) | 0.68(0.03) | 0.437 | 0.642 |
| Medium | 0.87(0.06) | 0.92(0.05) | 0.83(0.04) | 0.88(0.05) | 0.71(0.04) | 0.67(0.04) | 0.73(0.05) | <0.001 | 0.524 |
| High | 1.29(0.09) | 1.37(0.08) | 1.21(0.06) | 1.36(0.06) | 1.18(0.05) | 1.11(0.08) | 0.92(0.06) | <0.001 | 0.025 |
| **Total arsenic (ug/L)^1^** |  |  |  |  |  |  |  |  |  |
| Age group, y |  |  |  |  |  |  |  |  |  |
| 20–39 y | 9.17(1.03) | 9.73(1.06) | 9.21(1.02) | 9.47(1.11) | 7.12(0.65) | 6.61(0.47) | 5.69(0.42) | <0.001 | 0.011 |
| 40–59 y | 7.79(0.87) | 9.87(0.76) | 8.53(0.65) | 10.87(1.21) | 6.55(0.78) | 5.15(0.52) | 6.58(0.63) | 0.001 | 0.028 |
| ≥60 y | 8.44(0.56) | 9.85(0.85) | 7.28(0.78) | 10.13(1.08) | 8.00(0.69) | 6.95(0.67) | 7.00(0.75) | 0.006 | 0.129 |
| Sex |  |  |  |  |  |  |  |  |  |
| Male | 9.88(1.02) | 10.68(1.21) | 9.69(0.98) | 11.26(1.32) | 7.93(0.87) | 7.03(0.67) | 7.08(0.67) | <0.001 | 0.022 |
| Female | 7.27(0.87) | 9.07(0.87) | 7.37(0.76) | 9.21(0.87) | 6.36(0.76) | 5.98(0.65) | 5.68(0.64) | <0.001 | 0.011 |
| Race-ethnicity |  |  |  |  |  |  |  |  |  |
| NHW | 7.15(0.54) | 9.77(0.89) | 7.20(0.78) | 8.80(0.86) | 5.99(0.67) | 5.91(0.66) | 5.35(0.62) | <0.001 | 0.005 |
| NHB | 12.69(1.34) | 11.98(1.34) | 11.38(1.21) | 12.64(1.27) | 10.15(1.26) | 8.62(0.86) | 8.67(0.76) | <0.001 | 0.299 |
| MA | 10.28(1.03) | 10.06(1.11) | 9.79(0.98) | 9.43(0.89) | 7.62(0.85) | 5.85(0.67) | 6.61(0.46) | <0.001 | 0.235 |
| Socioeconomic status |  |  |  |  |  |  |  |  |  |
| Low | 7.93(0.46) | 10.69(1.12) | 7.83(0.56) | 8.90(0.62) | 6.79(0.48) | 5.48(0.37) | 7.11(0.47) | 0.007 | 0.639 |
| Medium | 8.98(0.56) | 9.34(0.63) | 8.37(0.52) | 8.99(0.47) | 6.74(0.34) | 6.18(0.46) | 6.44(0.32) | <0.001 | 0.299 |
| High | 7.81(0.35) | 10.23(1.21) | 8.61(0.56) | 11.73(0.97) | 7.24(0.72) | 7.11(0.68) | 5.88(0.63) | <0.001 | <0.001 |
| **Iodine (ng/mL)^1^** |  |  |  |  |  |  |  |  |  |
| Age group, y |  |  |  |  |  |  |  |  |  |
| 20–39 y | 136.55(7.16) | 129.78(6.50) | 153.50(5.50) | 127.91(7.88) | 118.80(8.98) | 118.58(6.28) | 104.14(4.95) | <0.001 | 0.001 |
| 40–59 y | 132.43(7.50) | 141.38(7.45) | 143.42(6.14) | 132.19(6.05) | 123.34(8.89) | 117.03(8.75) | 126.79(9.53) | 0.01 | 0.361 |
| ≥60 y | 172.46(10.22) | 203.28(17.53) | 179.39(7.61) | 162.82(12.17) | 157.80(11.73) | 162.67(10.42) | 157.07(10.46) | 0.004 | 0.876 |
| Sex |  |  |  |  |  |  |  |  |  |
| Male | 159.18(6.23) | 164.59(8.00) | 169.28(3.76) | 149.18(7.13) | 146.02(6.32) | 139.38(8.69) | 131.73(3.77) | <0.001 | 0.013 |
| Female | 126.77(4.61) | 136.05(5.61) | 142.63(5.45) | 127.79(4.91) | 114.71(4.93) | 117.79(4.00) | 118.19(6.89) | <0.001 | 0.098 |
| Race-ethnicity |  |  |  |  |  |  |  |  |  |
| NHW | 147.06(3.51) | 152.23(6.65) | 156.28(5.36) | 138.52(7.30) | 128.62(6.45) | 133.97(6.97) | 124.28(6.40) | <0.001 | 0.101 |
| NHB | 125.57(7.15) | 139.35(8.20) | 138.64(6.72) | 129.75(6.24) | 130.59(9.37) | 134.74(9.57) | 137.63(7.16) | 0.84 | 0.756 |
| MA | 160.51(7.15) | 163.61(8.37) | 161.70(7.93) | 138.52(7.30) | 141.35(12.31) | 118.41(6.88) | 124.22(10.33) | <0.001 | 0.477 |
| Socioeconomic status |  |  |  |  |  |  |  |  |  |
| Low | 151.25(6.75) | 144.31(7.06) | 161.99(5.00) | 134.56(6.56) | 141.81(6.55) | 134.15(6.59) | 132.13(6.65) | 0.035 | 0.389 |
| Medium | 150.24(8.53) | 158.82(10.09) | 158.42(4.48) | 141.61(7.70) | 130.74(8.58) | 131.86(7.80) | 127.94(7.62) | <0.001 | 0.212 |
| High | 132.89(7.64) | 139.64(6.77) | 147.43(6.29) | 129.03(5.24) | 121.20(7.76) | 123.68(8.21) | 114.79(7.24) | <0.001 | 0.032 |
|  | **Protein** | | | | | | |  |  |
| **Total protein (g/L)** |  |  |  |  |  |  |  |  |  |
| Age group, y |  |  |  |  |  |  |  |  |  |
| 20–39 y | 72.87(0.21) | 71.55(0.21) | 72.22(0.16) | 72.04(0.16) | 72.14(0.24) | 71.48(0.18) | 72.41(0.23) | 0.13 | 0.007 |
| 40–59 y | 71.66(0.28) | 70.68(0.26) | 71.24(0.14) | 71.16(0.22) | 70.71(0.27) | 70.06(0.25) | 71.14(0.22) | 0.01 | 0.102 |
| ≥60 y | 70.80(0.24) | 70.20(0.27) | 70.39(0.17) | 70.32(0.19) | 69.88(0.18) | 69.06(0.22) | 69.96(0.27) | <0.001 | 0.308 |
| Sex |  |  |  |  |  |  |  |  |  |
| Male | 72.52(0.25) | 71.39(0.25) | 71.79(0.16) | 71.74(0.20) | 71.35(0.24) | 70.71(0.22) | 71.80(0.23) | 0.002 | 0.009 |
| Female | 71.35(0.21) | 70.44(0.22) | 71.01(0.14) | 70.83(0.18) | 70.68(0.24) | 69.91(0.20) | 70.73(0.24) | 0.005 | 0.284 |
| Race-ethnicity |  |  |  |  |  |  |  |  |  |
| NHW | 71.17(0.19) | 70.23(0.27) | 70.71(0.09) | 70.46(0.17) | 70.24(0.20) | 69.35(0.17) | 70.59(0.22) | <0.001 | 0.016 |
| NHB | 73.71(0.20) | 72.72(0.26) | 73.26(0.25) | 73.03(0.20) | 72.74(0.16) | 72.08(0.20) | 72.71(0.24) | 0.003 | 0.190 |
| MA | 74.19(0.26) | 73.13(0.36) | 73.38(0.36) | 73.20(0.14) | 72.89(0.24) | 72.42(0.35) | 72.98(0.20) | <0.001 | 0.692 |
| Socioeconomic status |  |  |  |  |  |  |  |  |  |
| Low | 73.02(0.17) | 72.50(0.59) | 72.65(0.16) | 72.60(0.24) | 72.22(0.33) | 71.05(0.21) | 72.05(0.23) | <0.001 | 0.989 |
| Medium | 72.06(0.27) | 71.02(0.24) | 71.45(0.16) | 71.52(0.20) | 70.91(0.15) | 70.47(0.26) | 71.40(0.26) | 0.002 | 0.060 |
| High | 71.49(0.34) | 70.43(0.31) | 70.93(0.20) | 70.54(0.19) | 70.79(0.24) | 69.84(0.23) | 70.81(0.23) | 0.202 | 0.038 |
| **Albumin (g/L)** |  |  |  |  |  |  |  |  |  |
| Age group, y |  |  |  |  |  |  |  |  |  |
| 20–39 y | 43.76(0.16) | 42.97(0.15) | 43.41(0.13) | 43.62(0.15) | 44.03(0.13) | 43.66(0.08) | 44.57(0.13) | <0.001 | 0.001 |
| 40–59 y | 42.49(0.13) | 42.37(0.18) | 42.44(0.08) | 42.75(0.12) | 42.97(0.15) | 42.55(0.13) | 43.49(0.16) | <0.001 | 0.044 |
| ≥60 y | 41.55(0.14) | 41.61(0.21) | 41.72(0.07) | 41.67(0.12) | 42.13(0.13) | 41.90(0.09) | 42.79(0.08) | <0.001 | 0.001 |
| Sex |  |  |  |  |  |  |  |  |  |
| Male | 43.85(0.12) | 43.28(0.12) | 43.60(0.09) | 43.75(0.14) | 44.04(0.13) | 43.73(0.10) | 44.62(0.15) | <0.001 | <0.001 |
| Female | 41.70(0.13) | 41.57(0.16) | 41.69(0.08) | 41.89(0.13) | 42.27(0.10) | 41.87(0.08) | 42.77(0.13) | <0.001 | 0.013 |
| Race-ethnicity |  |  |  |  |  |  |  |  |  |
| NHW | 42.91(0.13) | 42.62(0.12) | 42.71(0.09) | 42.99(0.15) | 43.33(0.11) | 42.89(0.11) | 44.02(0.14) | <0.001 | <0.001 |
| NHB | 41.12(0.18) | 40.77(0.15) | 41.32(0.14) | 41.41(0.26) | 41.70(0.12) | 41.35(0.11) | 42.20(0.13) | <0.001 | 0.232 |
| MA | 43.18(0.29) | 42.36(0.27) | 43.11(0.17) | 42.71(0.10) | 43.20(0.20) | 43.06(0.12) | 44.12(0.22) | 0.004 | 0.420 |
| Socioeconomic status |  |  |  |  |  |  |  |  |  |
| Low | 42.01(0.12) | 41.90(0.09) | 42.04(0.10) | 41.87(0.20) | 42.68(0.23) | 42.09(0.13) | 42.74(0.17) | <0.001 | 0.342 |
| Medium | 42.63(0.12) | 42.22(0.17) | 42.52(0.11) | 42.66(0.12) | 42.93(0.08) | 42.52(0.11) | 43.48(0.13) | <0.001 | 0.003 |
| High | 43.12(0.12) | 42.74(0.22) | 42.80(0.10) | 43.19(0.13) | 43.51(0.11) | 43.18(0.08) | 44.09(0.12) | <0.001 | 0.001 |
|  | **Lipids** | | | | | | |  |  |
| **Apo B (mg/dL)** |  |  |  |  |  |  |  |  |  |
| Age group, y |  |  |  |  |  |  |  |  |  |
| 20–39 y | — | 95.40(1.87) | 87.20(0.84) | 84.70(1.15) | 83.48(1.03) | 84.52(1.27) | 86.73(0.79) | <0.001 | <0.001 |
| 40–59 y | — | 104.99(1.63) | 98.65(1.20) | 95.31(1.20) | 95.96(1.41) | 95.97(1.44) | 100.73(1.17) | 0.018 | <0.001 |
| ≥60 y | — | 103.35(1.22) | 93.07(1.00) | 91.63(1.41) | 90.27(0.73) | 89.15(1.16) | 92.49(1.22) | <0.001 | <0.001 |
| Sex |  |  |  |  |  |  |  |  |  |
| Male | — | 103.76(1.41) | 94.89(0.75) | 92.30(0.79) | 90.95(1.14) | 91.72(0.94) | 95.38(1.20) | <0.001 | <0.001 |
| Female | — | 98.47(1.72) | 91.74(1.05) | 89.41(1.00) | 89.21(0.90) | 88.64(0.64) | 92.28(0.87) | <0.001 | <0.001 |
| Race-ethnicity |  |  |  |  |  |  |  |  |  |
| NHW | — | 101.37(1.51) | 93.28(0.80) | 90.27(1.11) | 90.21(1.19) | 90.42(0.87) | 94.04(0.91) | <0.001 | <0.001 |
| NHB | — | 95.84(0.96) | 89.42(2.07) | 90.59(1.33) | 87.23(1.32) | 85.32(0.98) | 90.89(1.87) | <0.001 | <0.001 |
| MA | — | 102.56(1.51) | 96.45(1.24) | 95.78(1.50) | 92.25(1.74) | 94.64(2.46) | 95.36(1.17) | 0.001 | 0.001 |
| Socioeconomic status |  |  |  |  |  |  |  |  |  |
| Low | — | 103.14(1.56) | 96.32(0.94) | 93.70(0.83) | 91.19(1.12) | 90.08(0.95) | 96.76(1.68) | <0.001 | <0.001 |
| Medium | — | 100.39(1.28) | 93.46(0.88) | 91.66(1.28) | 90.13(1.17) | 89.67(0.80) | 93.12(1.74) | <0.001 | <0.001 |
| High | — | 101.63(1.70) | 92.81(1.57) | 88.72(1.27) | 89.28(1.14) | 90.75(1.18) | 94.59(0.82) | 0.001 | <0.001 |
| **HDL-C (mg/dL)** |  |  |  |  |  |  |  |  |  |
| Age group, y |  |  |  |  |  |  |  |  |  |
| 20–39 y | 53.04(0.58) | 52.68(0.40) | 50.42(0.62) | 50.73(0.58) | 51.29(0.65) | 50.91(0.51) | 53.57(0.86) | 0.86 | <0.001 |
| 40–59 y | 53.68(0.65) | 54.66(0.48) | 52.00(0.65) | 54.08(0.50) | 52.45(0.74) | 53.56(0.52) | 54.83(0.91) | 0.65 | 0.057 |
| ≥60 y | 55.57(0.60) | 56.49(0.50) | 53.78(0.62) | 54.67(0.85) | 55.55(1.22) | 55.11(0.56) | 58.81(1.14) | 0.03 | 0.003 |
| Sex |  |  |  |  |  |  |  |  |  |
| Male | 48.32(0.46) | 48.61(0.32) | 46.33(0.47) | 47.70(0.45) | 47.80(0.46) | 47.64(0.38) | 49.15(0.69) | 0.41 | 0.005 |
| Female | 59.23(0.65) | 59.85(0.60) | 57.08(0.69) | 58.07(0.44) | 57.59(0.71) | 58.13(0.32) | 61.55(0.92) | 0.23 | <0.001 |
| Race-ethnicity |  |  |  |  |  |  |  |  |  |
| NHW | 54.01(0.50) | 54.49(0.40) | 51.89(0.71) | 53.45(0.56) | 53.31(0.60) | 53.52(0.33) | 56.75(0.75) | 0.02 | <0.001 |
| NHB | 56.41(0.77) | 57.10(0.42) | 56.71(0.53) | 54.95(0.65) | 54.67(0.47) | 54.89(0.62) | 58.23(0.57) | 0.89 | 0.001 |
| MA | 50.41(0.45) | 50.29(0.72) | 48.06(0.32) | 49.34(1.06) | 49.10(0.80) | 49.15(0.55) | 49.30(0.91) | 0.28 | 0.192 |
| Socioeconomic status |  |  |  |  |  |  |  |  |  |
| Low | 51.49(0.68) | 50.10(0.54) | 48.99(0.68) | 50.42(0.57) | 48.85(0.69) | 48.93(0.71) | 51.07(0.89) | 0.10 | 0.041 |
| Medium | 53.24(0.34) | 53.81(0.41) | 51.15(0.59) | 51.73(0.53) | 51.77(0.72) | 51.61(0.58) | 53.55(0.28) | 0.03 | <0.001 |
| High | 55.00(0.43) | 55.47(0.50) | 52.97(0.48) | 55.56(0.39) | 55.03(0.43) | 55.56(0.44) | 58.76(0.64) | 0.08 | 0.002 |
| **LDL-C (mg/dL)** |  |  |  |  |  |  |  |  |  |
| Age group, y |  |  |  |  |  |  |  |  |  |
| 20–39 y | 109.80(1.33) | 108.65(2.29) | 110.14(1.43) | 109.14(1.40) | 109.51(1.15) | 105.92(1.74) | 106.12(1.06) | 0.15 | 0.161 |
| 40–59 y | 121.87(2.18) | 121.12(1.64) | 123.17(1.52) | 123.03(1.58) | 122.70(1.66) | 119.15(1.57) | 122.43(1.98) | 0.53 | 0.616 |
| ≥60 y | 120.04(1.72) | 114.58(1.41) | 111.84(1.68) | 113.72(2.24) | 112.27(1.37) | 107.87(1.58) | 108.67(1.53) | <0.001 | 0.362 |
| Sex |  |  |  |  |  |  |  |  |  |
| Male | 117.62(1.33) | 116.14(1.39) | 116.06(0.89) | 116.39(1.16) | 113.61(1.24) | 111.65(1.32) | 113.51(1.55) | 0.001 | 0.900 |
| Female | 116.23(1.50) | 114.30(1.49) | 115.57(1.26) | 115.54(1.07) | 116.75(1.11) | 111.26(1.06) | 112.51(1.28) | 0.10 | 0.179 |
| Race-ethnicity |  |  |  |  |  |  |  |  |  |
| NHW | 118.23(1.31) | 115.67(1.28) | 115.82(0.94) | 115.97(1.42) | 115.43(1.32) | 111.76(1.20) | 113.47(1.35) | 0.002 | 0.963 |
| NHB | 114.66(1.62) | 110.78(1.89) | 112.66(2.13) | 115.73(0.95) | 113.05(1.57) | 109.77(1.63) | 110.49(2.81) | 0.30 | 0.440 |
| MA | 115.28(1.31) | 114.82(2.20) | 117.71(1.92) | 117.88(1.97) | 114.92(2.07) | 113.20(3.70) | 112.57(1.41) | 0.60 | 0.080 |
| Socioeconomic status |  |  |  |  |  |  |  |  |  |
| Low | 123.52(2.37) | 114.40(1.58) | 118.34(1.38) | 115.13(1.45) | 111.62(1.83) | 107.11(3.71) | 114.50(1.56) | 0.05 | 0.151 |
| Medium | 116.30(1.71) | 113.37(1.76) | 115.39(1.49) | 116.44(1.87) | 114.68(1.36) | 110.25(3.20) | 112.42(2.32) | 0.01 | 0.282 |
| High | 117.32(1.91) | 117.91(1.71) | 116.16(1.95) | 115.53(1.15) | 116.63(1.39) | 114.04(3.89) | 114.61(1.33) | 0.05 | 0.964 |
| **TC (mg/dL)** |  |  |  |  |  |  |  |  |  |
| Age group, y |  |  |  |  |  |  |  |  |  |
| 20–39 y | 190.02(1.06) | 189.48(1.13) | 187.95(1.29) | 184.62(0.87) | 183.22(1.16) | 180.67(1.72) | 181.28(0.95) | <0.001 | 0.609 |
| 40–59 y | 209.48(1.62) | 206.07(1.39) | 206.24(1.54) | 205.96(1.86) | 205.67(1.33) | 198.44(0.78) | 203.07(2.20) | <0.001 | 0.858 |
| ≥60 y | 207.00(1.14) | 200.01(1.32) | 196.09(1.16) | 196.91(1.76) | 196.29(1.74) | 188.24(1.47) | 192.43(1.29) | <0.001 | 0.015 |
| Sex |  |  |  |  |  |  |  |  |  |
| Male | 200.59(0.79) | 195.89(1.05) | 194.62(1.11) | 194.15(1.18) | 191.01(1.21) | 186.00(1.22) | 189.46(1.55) | <0.001 | 0.131 |
| Female | 202.51(1.36) | 201.14(0.91) | 199.59(1.20) | 197.72(1.13) | 199.21(1.20) | 192.48(0.84) | 195.17(1.41) | <0.001 | 0.906 |
| Race-ethnicity |  |  |  |  |  |  |  |  |  |
| NHW | 203.15(0.95) | 199.63(0.88) | 197.86(0.98) | 197.03(1.20) | 196.99(1.35) | 190.07(0.95) | 194.13(1.41) | <0.001 | 0.226 |
| NHB | 195.47(1.32) | 189.87(0.94) | 192.36(1.68) | 190.01(1.38) | 189.03(1.05) | 182.89(1.60) | 185.43(1.80) | <0.001 | 0.962 |
| MA | 196.49(1.53) | 197.77(1.64) | 197.21(1.93) | 196.76(0.96) | 193.52(1.81) | 190.28(2.30) | 191.57(1.55) | <0.001 | 0.154 |
| Socioeconomic status |  |  |  |  |  |  |  |  |  |
| Low | 207.38(1.82) | 196.93(1.03) | 196.17(1.36) | 193.46(1.09) | 191.44(1.41) | 184.73(1.29) | 189.79(1.94) | <0.001 | 0.039 |
| Medium | 199.76(1.77) | 197.39(1.21) | 197.17(1.01) | 195.37(1.82) | 193.15(1.63) | 188.20(1.42) | 189.90(2.12) | <0.001 | 0.699 |
| High | 203.25(1.51) | 200.38(1.22) | 197.24(1.56) | 196.81(1.07) | 198.75(1.10) | 192.31(0.92) | 196.77(1.95) | <0.001 | 0.137 |
| **TG (mg/dL)^1^** |  |  |  |  |  |  |  |  |  |
| Age group, y |  |  |  |  |  |  |  |  |  |
| 20–39 y | 107.15(3.53) | 105.29(3.22) | 100.02(4.32) | 98.65(2.26) | 97.45(3.75) | 88.14(3.29) | 99.83(0.83) | <0.001 | 0.567 |
| 40–59 y | 129.33(3.33) | 123.22(2.53) | 121.65(2.74) | 110.03(2.21) | 123.48(5.73) | 105.03(3.54) | 126.61(4.51) | <0.001 | 0.544 |
| ≥60 y | 138.58(3.64) | 128.68(3.58) | 125.17(3.57) | 117.00(2.66) | 116.36(5.16) | 102.59(4.12) | 112.94(4.83) | <0.001 | 0.559 |
| Sex |  |  |  |  |  |  |  |  |  |
| Male | 132.26(3.08) | 127.76(2.89) | 124.45(2.28) | 115.69(2.80) | 121.80(3.94) | 105.09(3.47) | 123.56(3.29) | <0.001 | 0.698 |
| Female | 113.71(3.54) | 107.76(2.42) | 105.56(2.72) | 100.93(2.28) | 103.09(3.08) | 92.19(2.42) | 104.88(2.53) | <0.001 | 0.927 |
| Race-ethnicity |  |  |  |  |  |  |  |  |  |
| NHW | 125.11(3.38) | 119.70(2.30) | 116.76(2.37) | 108.27(1.96) | 115.53(3.52) | 99.95(2.62) | 115.49(2.16) | <0.001 | 0.923 |
| NHB | 99.97(2.46) | 95.07(2.72) | 85.22(4.47) | 88.01(2.73) | 85.95(2.69) | 77.07(3.05) | 88.83(5.64) | <0.001 | 0.422 |
| MA | 130.75(6.77) | 125.57(6.36) | 126.72(4.60) | 123.15(4.58) | 118.87(4.28) | 112.79(3.90) | 124.89(1.49) | 0.005 | 0.616 |
| Socioeconomic status |  |  |  |  |  |  |  |  |  |
| Low | 136.24(4.17) | 128.50(1.41) | 122.09(2.21) | 126.42(1.46) | 120.53(2.61) | 110.79(3.71) | 102.94(2.23) | <0.001 | 0.417 |
| Medium | 123.00(3.27) | 118.14(3.38) | 116.17(3.26) | 110.58(2.34) | 113.53(2.79) | 98.26(3.20) | 92.47(1.33) | <0.001 | 0.441 |
| High | 120.61(4.07) | 114.71(4.59) | 112.02(4.23) | 98.33(3.64) | 108.27(5.83) | 95.98(3.89) | 96.81(2.77) | <0.001 | 0.160 |

Abbreviation: SE, Standard Error; NHW, non-Hispanic white; NHB, non-Hispanic black; MA, Mexican American, 25(OH)D, 25-hydroxyvitamin D; Apo B, apolipoprotein B; HDL-C, High-Density Lipoprotein Cholesterol; LDL-C, Low-Density Lipoprotein Cholesterol; TC, Total Cholesterol; TG, Triglyceride.

^1^Geometric mean

^2^ Calculated by using linear regression model.

**Supplementary Table 7**

Mean (SE) of nutritional biomarkers by demographic variables in the National Health and Nutrition Examination Survey (2003-2016) ^a^

|  | Vitamin B_6_ | Serum folate | RBC folate | Vitamin D | Cadmium | Lead | Mercury | Phosphorus | Potassium |
| --- | --- | --- | --- | --- | --- | --- | --- | --- | --- |
| Age group, y |  |  |  |  |  |  |  |  |  |
| 20–39 y | 47.53(1.23) | 14.88(0.23) | 436.21(8.96) | 62.74(0.66) | 0.30(0.01) | 0.92(0.02) | 0.81(0.05) | 1.23(0.00) | 3.92(0.01) |
| 40–59 y | 48.07(1.65) | 16.49(0.32) | 487.30(9.87) | 66.34(0.69) | 0.37(0.02) | 1.36(0.03) | 0.99(0.06) | 1.21(0.00) | 3.97(0.01) |
| ≥60 y | 51.14(2.25) | 21.31(0.34) | 581.17(13.29) | 71.96(0.66) | 0.41(0.01) | 1.71(0.03) | 1.05(0.06) | 1.21(0.00) | 4.07(0.01) |
| *P* value | <0.001 | <0.001 | <0.001 | <0.001 | <0.001 | <0.001 | <0.001 | <0.001 | <0.001 |
| Sex |  |  |  |  |  |  |  |  |  |
| Male | 54.44(2.78) | 15.88(0.30) | 477.42(8.92) | 64.75(0.57) | 0.32(0.01) | 1.48(0.02) | 0.98(0.05) | 1.19(0.00) | 4.03(0.01) |
| Female | 43.61(1.21) | 18.04(0.32) | 501.42(9.33) | 68.04(0.65) | 0.38(0.01) | 1.06(0.01) | 0.91(0.05) | 1.24(0.00) | 3.93(0.01) |
| *P* value | <0.001 | <0.001 | <0.001 | <0.001 | <0.001 | <0.001 | <0.001 | <0.001 | <0.001 |
| Race-ethnicity |  |  |  |  |  |  |  |  |  |
| NHW | 50.99(1.97) | 14.94(0.29) | 522.04(9.98) | 72.55(0.57) | 0.35(0.02) | 1.24(0.03) | 0.91(0.06) | 1.22(0.001) | 4.00(0.01) |
| NHB | 37.25(0.77) | 13.54(0.27) | 394.91(7.98) | 45.61(0.67) | 0.40(0.03) | 1.32(0.00) | 0.99(0.06) | 1.21(0.001) | 3.92(0.01) |
| MA | 45.23(1.34) | 18.03(0.33) | 438.83(8.65) | 53.72(0.73) | 0.29(0.01) | 1.25(0.03) | 0.68(0.04) | 1.21(0.001) | 3.93(0.01) |
| *P* value | <0.001 | <0.001 | <0.001 | <0.001 | <0.001 | <0.001 | <0.001 | <0.001 | <0.001 |
| Socioeconomic status |  |  |  |  |  |  |  |  |  |
| Low | 34.51(0.75) | 14.50(0.30) | 435.51(9.87) | 58.31(1.09) | 0.47(0.05) | 1.53(0.04) | 1.23(0.07) | 1.21(0.001) | 3.97(0.01) |
| Medium | 44.43(1.34) | 16.59(0.32) | 483.95(10.96) | 64.17(0.61) | 0.38(0.03) | 1.24(0.02) | 0.81(0.05) | 1.22(0.001) | 3.97(0.01) |
| High | 58.21(1.89) | 18.06(0.35) | 511.33(11.65) | 71.04(0.67) | 0.30(0.01) | 1.19(0.01) | 0.66(0.04) | 1.22(0.001) | 3.99(0.01) |
| *P* value | <0.001 | <0.001 | <0.001 | <0.001 | <0.001 | <0.001 | <0.001 | 0.286 | <0.001 |
|  | Iodine | Sodium | Total protein | Albumin | Apo B | HDL-C | LDL-C | TC | TG |
| Age group, y |  |  |  |  |  |  |  |  |  |
| 20–39 y | 133.16(2.58) | 139.05(0.05) | 72.11(0.08) | 43.73(0.05) | 86.95(0.50) | 51.79(0.24) | 108.57(0.58) | 185.18(0.46) | 96.65(2.34) |
| 40–59 y | 131.23(3.21) | 139.10(0.05) | 70.94(0.09) | 42.73(0.06) | 98.51(0.57) | 53.61(0.25) | 121.93(0.66) | 204.93(0.59) | 116.28(3.32) |
| ≥60 y | 171.04(5.64) | 139.38(0.06) | 70.04(0.09) | 41.95(0.05) | 92.94(0.53) | 55.80(0.34) | 112.25(0.65) | 196.13(0.57) | 115.64(3.43) |
| *P* value | <0.001 | <0.001 | <0.001 | <0.001 | <0.001 | <0.001 | <0.001 | <0.001 | <0.001 |
| Sex |  |  |  |  |  |  |  |  |  |
| Male | 153.96(3.87) | 139.29(0.05) | 71.60(0.09) | 43.85(0.05) | 94.74(0.46) | 47.94(0.18) | 114.90(0.49) | 192.91(0.45) | 117.63(4.32) |
| Female | 129.06(2.51) | 139.03(0.05) | 70.69(0.08) | 41.98(0.05) | 91.48(0.45) | 58.80(0.26) | 114.57(0.48) | 188.10(0.44) | 101.18(2.78) |
| *P* value | <0.001 | <0.001 | <0.001 | <0.001 | <0.001 | <0.001 | 0.583 | <0.001 | <0.001 |
| Race-ethnicity |  |  |  |  |  |  |  |  |  |
| NHW | 142.92(3.78) | 139.02(0.06) | 70.39(0.08) | 43.07(0.05) | 93.21(0.49) | 53.92(0.24) | 115.16(0.49) | 194.68(0.45) | 111.46(3.65) |
| NHB | 134.83(4.65) | 139.17(0.06) | 72.94(0.09) | 41.43(0.07) | 89.90(0.63) | 56.13(0.24) | 112.41(0.77) | 189.10(0.59) | 85.62(2.18) |
| MA | 145.21(3.98) | 139.26(0.06) | 72.98(0.11) | 42.97(0.07) | 95.98(0.71) | 49.34(0.29) | 115.21(0.94) | 196.94(0.73) | 119.62(3.98) |
| *P* value | 0.045 | <.001 | <0.001 | <0.001 | <0.001 | <0.001 | 0.014 | <0.001 | <0.001 |
| Socioeconomic status |  |  |  |  |  |  |  |  |  |
| Low | 147.16(4.56) | 139.01(0.06) | 72.29(0.12) | 42.19(0.08) | 94.61(0.78) | 49.91(0.35) | 114.68(0.93) | 194.11(0.89) | 120.67(3.87) |
| Medium | 144.91(4.34) | 139.15(0.06) | 71.25(0.10) | 42.72(0.05) | 92.96(0.52) | 52.41(0.19) | 114.06(0.51) | 194.23(0.41) | 109.60(2.79) |
| High | 132.89(3.78) | 139.19(0.06) | 70.68(0.08) | 43.24(0.05) | 93.04(0.60) | 55.52(0.29) | 116.00(0.70) | 197.85(0.63) | 106.19(2.60) |
| *P* value | <0.001 | <0.001 | <0.001 | <0.001 | <0.001 | <0.001 | 0.006 | <0.001 | <0.001 |

Abbreviation: HDL-C, High-Density Lipoprotein Cholesterol; LDL-C, Low-density Lipoprotein Cholesterol; TC, Total Cholesterol; TG, Triglyceride.

^a^ *P* value were calculated by using analysis of variance (ANOVA), then Student-Newman-Keuls (SNK) were used for multiple comparisons.

**Supplementary Table 8**

Trends of nutritional biomarkers among US adults by dietary supplements use in the National Health and Nutrition Examination Survey (2003-2016).

| **Characteristics** | **2003-2004** | **2005-2006** | **2007-2008** | **2009-2010** | **2011-2012** | **2013-2014** | **2015-2016** | ***P*-linear** | ***P-*quadratic** |
| --- | --- | --- | --- | --- | --- | --- | --- | --- | --- |
| Plasma vitamin B_6_ (nmol/L) |  |  |  |  |  |  |  |  |  |
| Use | 91.82(5.62) | 102.24(3.84) | 105.70(4.53) | 96.34((3.20) | — | — | — | 0.008 | <0.001 |
| Not use | 44.59(2.06) | 46.45(1.66) | 47.00(1.43) | 46.73(1.82) | — | — | — | 0.044 | 0.029 |
| Serum folate (ng/mL) |  |  |  |  |  |  |  |  |  |
| Use | 23.57(0.45) | 23.20(0.59) | 24.82(0.41) | 23.81(0.59) | 23.64(0.52) | 24.03(0.31) | 23.98(1.03) | 0.020 | 0.449 |
| Not use | 14.98(0.20) | 15.71(0.32) | 14.49(0.31) | 14.38(0.14) | 15.90(0.38) | 15.83(0.21) | 14.81(0.22) | 0.884 | 0.206 |
| RBC folate(ng/mL)^1^ |  |  |  |  |  |  |  |  |  |
| Use | 598.24(9.32) | 627.81(9.51) | 646.39(12.99) | 593.36(1.25) | 584.50(8.89) | 635.93(8.95) | 623.48(12.26) | 0.152 | 0.693 |
| Not use | 435.24(4.18) | 463.28(7.06) | 457.89(8.19) | 431.26(5.81) | 438.68(8.35) | 470.75(6.16) | 464.88(8.24) | 0.002 | 0.206 |
| Vitamin B_12_ (pg/mL)^1^ |  |  |  |  |  |  |  |  |  |
| Use | 574.01(17.62) | 604.35(19.54) | — | — | 702.02(21.63) | 720.70(16.04) | — | <0.001 | 0.261 |
| Not use | 474.02(13.77) | 490.62(15.66) | — | — | 518.93(5.52) | 512.87(9.59) | — | <0.001 | 0.142 |
| 25-hydroxyvitamin D (nmol/L) |  |  |  |  |  |  |  |  |  |
| Use | 65.94(1.46) | 64.37(1.03) | 73.51(0.56) | 75.88(1.39) | 80.60(1.43) | 79.39(1.24) | — | <0.001 | 0.186 |
| Not use | 58.10(1.97) | 56.30(1.37) | 60.87(1.84) | 59.31(1.46) | 60.00(1.93) | 60.91(1.12) | — | 0.544 | 0.259 |
|  |  |  |  |  |  |  |  |  |  |
| Blood lead (ug/dL) |  |  |  |  |  |  |  |  |  |
| Use | 4.66(0.09) | 4.36(0.15) | 4.16(0.20) | 4.16(0.08) | 4.20(0.13) | 3.69(0.13) | 3.59(0.16) | <0.001 | 0.202 |
| Not use | 5.62(0.18) | 5.53(0.26) | 5.50(0.27) | 4.82(0.16) | 5.02(0.27) | 4.12(0.24) | 4.17(0.13) | <0.001 | 0.055 |
| Blood cadmium (ug/L) |  |  |  |  |  |  |  |  |  |
| Use | 0.09(0.00) | 0.08(0.00) | 0.07(0.00) | 0.07(0.00) | 0.07(0.00) | 0.06(0.00) | 0.05(0.00) | <0.001 | 0.049 |
| Not use | 0.10(0.00) | 0.09(0.00) | 0.09(0.00) | 0.08(0.00) | 0.07(0.00) | 0.06(0.00) | 0.06(0.00) | <0.001 | 0.002 |
| Blood total mercury (ug/L) |  |  |  |  |  |  |  |  |  |
| Use | 7.23(0.52) | 7.19(0.32) | 7.26(0.70) | 8.11(0.42) | 6.58(0.51) | 6.10(0.40) | 6.25(0.54) | 0.002 | 0.045 |
| Not use | 9.33(0.57) | 9.58(0.43) | 8.67(0.60) | 9.52(0.53) | 8.81(1.17) | 7.95(0.40) | 7.49(0.49) | <0.001 | 0.152 |
| Phosphorus (mmol/L) |  |  |  |  |  |  |  |  |  |
| Use | 1.23(0.00) | 1.24(0.00) | 1.23(0.01) | 1.23(0.00) | 1.21(0.00) | 1.24(0.01) | 1.20(0.01) | 0.003 | 0.112 |
| Not use | 1.22(0.01) | 1.23(0.00) | 1.21(0.00) | 1.20(0.01) | 1.20(0.00) | 1.23(0.01) | 1.18(0.01) | 0.002 | 0.733 |
| Total calcium (mmo/L) |  |  |  |  |  |  |  |  |  |
| Use | 2.39(0.00) | 2.38(0.00) | 2.35(0.01) | 2.37(0.00) | 2.35(0.00) | 2.37(0.00) | 2.34(0.00) | <0.001 | 0.025 |
| Not use | 2.39(0.00) | 2.37(0.00) | 2.35(0.01) | 2.36(0.00) | 2.23(0.00) | 2.36(0.00) | 2.34(0.00) | <0.001 | 0.006 |
| Urinary iodine (ng/mL) |  |  |  |  |  |  |  |  |  |
| Use | 210.76(6.57) | 262.25(10.57) | 241.07(7.02) | 225.69(7.48) | 191.70(11.90) | 192.19(9.22) | 202.80(9.36) | <0.001 | 0.042 |
| Not use | 213.42(6.32) | 285.75(7.08) | 242.35(6.16) | 200.99(5.73) | 264.95(8.97) | 201.38(8.92) | 258.77(7.45) | 0.001 | 0.061 |
| Urinary total arsenic (ug/L) |  |  |  |  |  |  |  |  |  |
| Use | 18.20(2.32) | 22.40(3.17) | 18.03(2.27) | 24.95(3.79) | 14.87(0.96) | 14.80(1.36) | 13.80(1.30) | 0.001 | 0.001 |
| Not use | 21.53(2.59) | 20.72(2.30) | 19.57(3.54) | 26.99(2.27) | 14.96(1.58) | 12.97(1.56) | 14.64(1.82) | 0.001 | 0.060 |
|  |  |  |  |  |  |  |  |  |  |
| Total protein (g/L) |  |  |  |  |  |  |  |  |  |
| Use | 71.55(0.20) | 70.56(0.27) | 70.95(0.13) | 70.81(0.17) | 70.68(0.23) | 69.70(0.22) | 70.72(0.22) | <0.001 | 0.058 |
| Not use | 72.35(0.28) | 71.30(0.20) | 71.82(0.17) | 71.72(0.20) | 71.36(0.26) | 71.00(0.19) | 71.93(0.27) | 0.037 | 0.075 |
| Albumin (g/L) |  |  |  |  |  |  |  |  |  |
| Use | 42.79(0.12) | 42.54(0.15) | 42.56(0.09) | 42.78(0.14) | 43.11(0.13) | 42.72(0.11) | 43.61(0.13) | <0.001 | 0.001 |
| Not use | 42.70(0.16) | 42.26(0.17) | 42.69(0.08) | 42.81(0.13) | 43.15(0.10) | 42.83(0.09) | 43.75(0.15) | <0.001 | 0.008 |
|  |  |  |  |  |  |  |  |  |  |
| Apo B (mg/dL) |  |  |  |  |  |  |  |  |  |
| Use | — | 100.99(1.39) | 93.19(0.87) | 90.89(0.93) | 89.08(0.88) | 90.14(0.90) | 92.83(0.86) | <0.001 | <0.001 |
| Not use | — | 101.35(1.63) | 93.42(0.76) | 90.65(0.88) | 91.10(1.28) | 90.22(0.93) | 95.04(1.17) | 0.001 | <0.001 |
| HDL-C (mg/dL) |  |  |  |  |  |  |  |  |  |
| Use | 55.84(0.45) | 56.05(0.41) | 54.19(0.45) | 55.54(0.62) | 55.00(0.56) | 55.09(0.43) | 57.49(0.88) | 0.241 | 0.006 |
| Not use | 51.49(0.49) | 52.38(0.50) | 49.57(0.55) | 50.52(0.40) | 50.51(0.51) | 50.64(0.38) | 53.09(0.76) | 0.392 | <0.001 |
| LDL-C (mg/dL) |  |  |  |  |  |  |  |  |  |
| Use | 117.47(1.88) | 115.52(1.43) | 114.86(1.12) | 116.21(1.18) | 114.24(1.08) | 111.50(1.04) | 111.65(1.28) | 0.001 | 0.643 |
| Not use | 116.45(1.31) | 114.90(1.70) | 116.89(0.94) | 115.67(0.97) | 116.32(1.32) | 111.53(1.18) | 114.82(1.66) | 0.046 | 0.570 |
| TC (mg/dL) |  |  |  |  |  |  |  |  |  |
| Use | 203.27(1.33) | 200.45(1.01) | 198.01(1.04) | 197.64(0.95) | 196.67(1.43) | 190.67(0.82) | 193.64(0.67) | <0.001 | 0.472 |
| Not use | 199.01(1.04) | 196.44(0.98) | 196.43(0.81) | 194.30(1.19) | 193.68(0.92) | 187.87(1.25) | 190.77(1.24) | <0.001 | 0.915 |
| TG (mg/dL) |  |  |  |  |  |  |  |  |  |
| Use | 154.33(6.72) | 141.29(4.20) | 136.12(3.51) | 123.20(2.72) | 130.06(3.08) | 123.96(3.71) | 111.41(2.40) | <0.001 | 0.303 |
| Not use | 142.85(4.64) | 142.06(4.57) | 138.03(2.77) | 130.87(3.13) | 134.91(4.36) | 117.84(3.28) | 116.62(3.61) | <0.001 | 0.198 |

**Supplementary Table 9**

The differences of mean concentrations of nutritional biomarkers among participants with and without dietary supplementary in NAHENS 2003-2016

|  | With supplements | Without supplements | *P* value |
| --- | --- | --- | --- |
| Plasma vitamin B_6_ (nmol/L) | 98.93(2.27) | 46.23(0.88) | <0.001 |
| Serum folate (ng/mL) | 23.87(0.24) | 15.15(0.10) | <0.001 |
| RBC folate(ng/mL) | 616.02(4.31) | 451.67(2.76) | <0.001 |
| Vitamin B_12_ (pg/mL) | 652.94(9.66) | 500.17(6.41) | <0.001 |
| 25-hydroxyvitamin D (nmol/L) | 73.45(0.57) | 58.76(0.68) | <0.001 |
| Blood lead (ug/dL) | 4.18(0.05) | 5.09(0.09) | <0.001 |
| Blood cadmium (ug/L) | 0.07(0.00) | 0.08(0.00) | <0.001 |
| Blood total mercury (ug/L) | 7.09(0.20) | 8.90(0.27) | <0.001 |
| Phosphorus(mmol/L) | 1.22(0.00) | 1.21(0.00) | <0.001 |
| Total calcium(mmo/L) | 2.36(0.00) | 2.36(0.00) | <0.001 |
| Urinary iodine(ng/mL) | 268.48(9.34) | 238.57(10.64) | 0.005 |
| Urinary total arsenic(ug/L) | 18.37(0.89) | 18.73(0.88) | 0.761 |
| Total protein(g/L) | 70.69(0.08) | 71.63(0.08) | <0.001 |
| Albumin (g/L) | 42.89(0.50) | 42.89(0.05) | 0.760 |
| Apo B(mg/dL) | 92.80(0.43) | 93.37(0.48) | 0.339 |
| HDL-C(mg/dL) | 55.64(0.23) | 51.13(0.21) | <0.001 |
| LDL-C(mg/dL) | 114.34(0.51) | 115.22(0.49) | 0.554 |
| TC (mg/dL) | 197.02(0.48) | 193.96(0.41) | <0.001 |
| TG (mg/dL) | 130.99(1.74) | 131.74(1.43) | 0.352 |


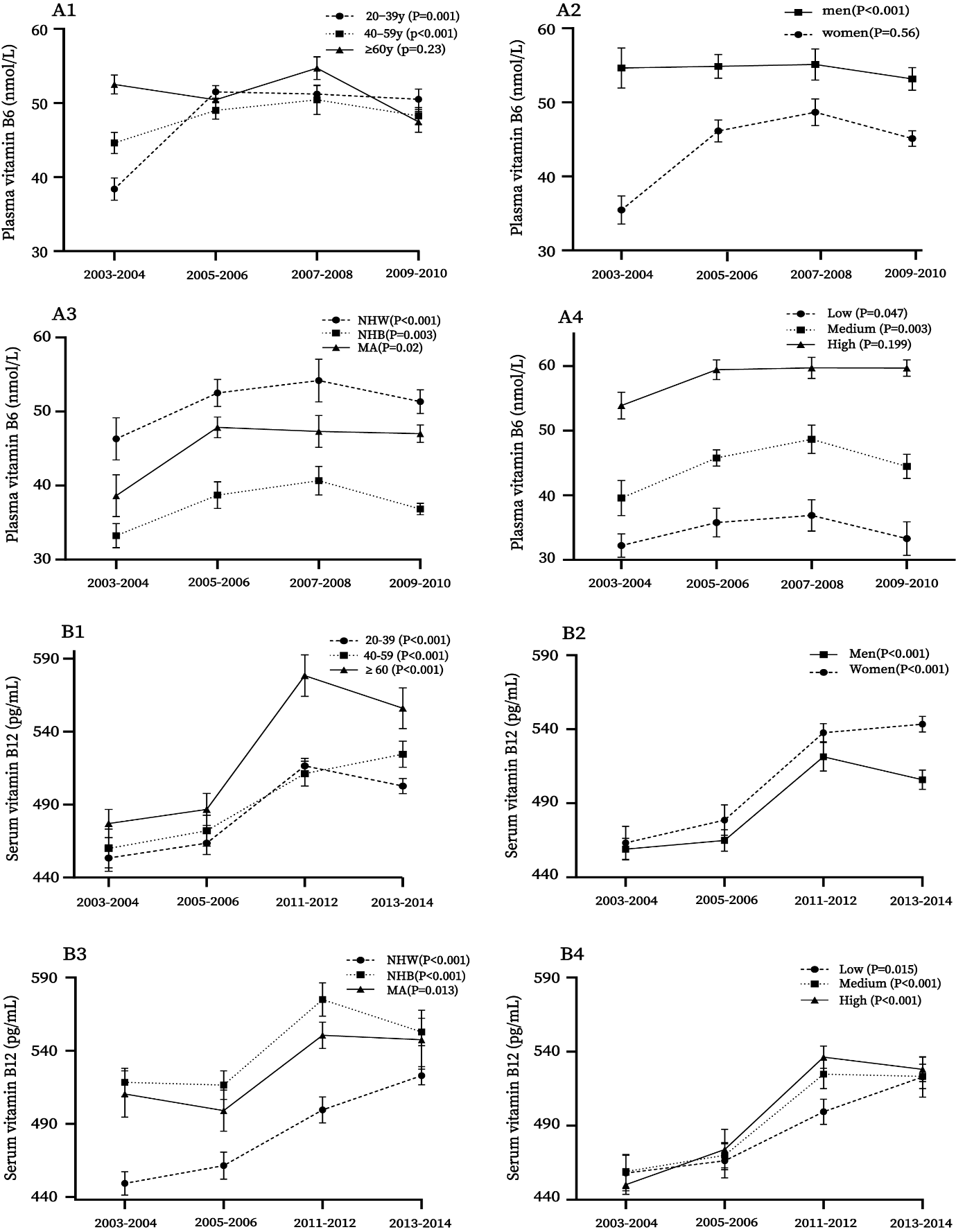


**Supplementary Figure 1** Trends of concentrations of serum vitamin B_6_ (A1-A4) and vitamin B_12_ (B1-B4) by age, sex, race-ethnicity and socioeconomic status (SS) among US adults from 2003 to 2016. MA, Mexican American; NHW, non-Hispanic white; NHB non-Hispanic black.


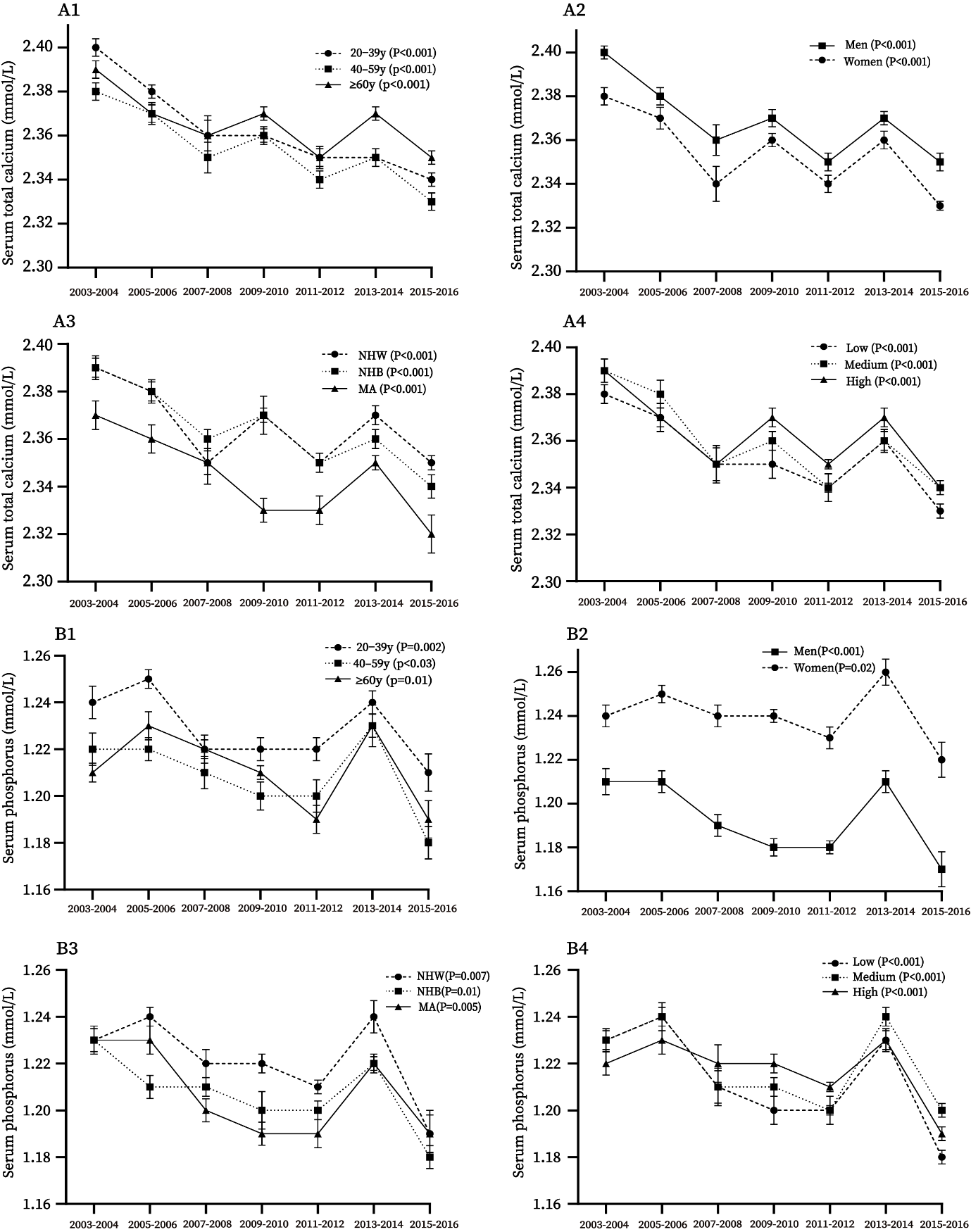


**Supplementary Figure 2** Trends of concentrations of serum calcium (A1-A4) and phosphorus (B1-B4) by age, sex, race-ethnicity and socioeconomic status (SS) among US adults from 2003 to 2016. MA, Mexican American; NHW, non-Hispanic white; NHB non-Hispanic black.


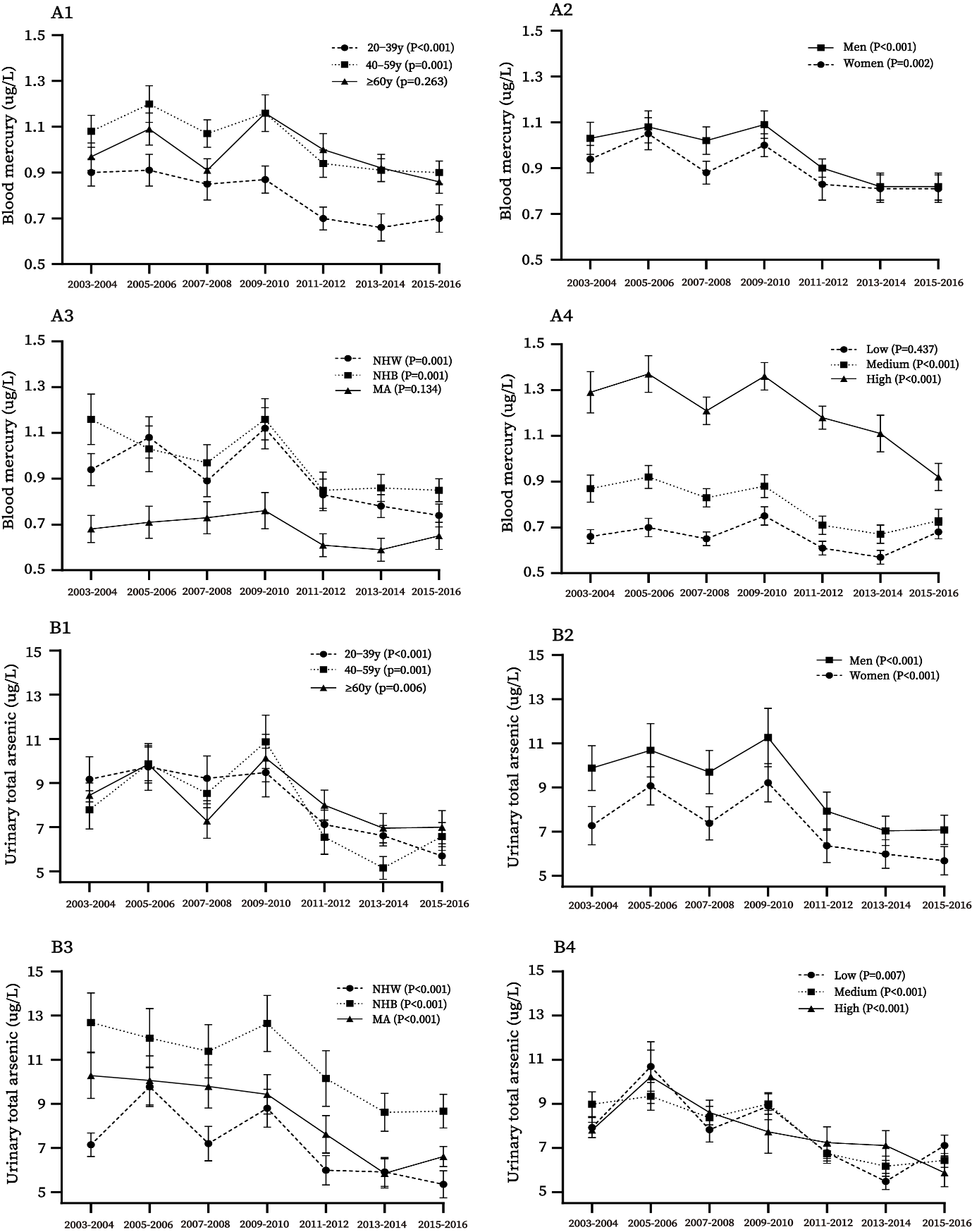


**Supplementary Figure 3** Trends of concentrations of blood mercury (A1-A4) and urinary arsenic (B1-B4) by age, sex, race-ethnicity and socioeconomic status (SS) among US adults from 2003 to 2016. MA, Mexican American; NHW, non-Hispanic white; NHB non-Hispanic black.


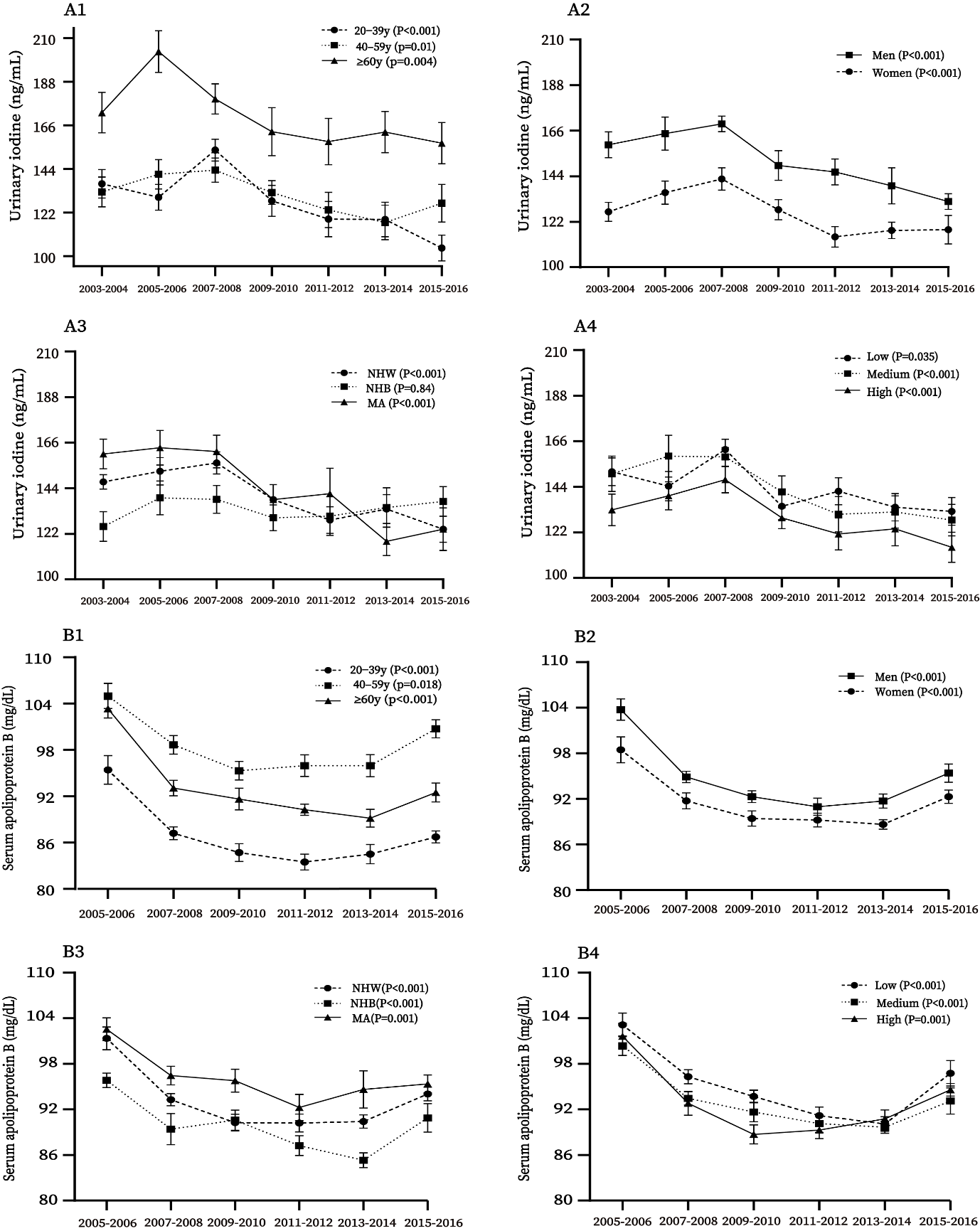


**Supplementary Figure 4** Trends of concentrations of urinary iodine (A1-A4) and serum Apo B (B1-B4) by age, sex, race-ethnicity and socioeconomic status (SS) among US adults from 2003 to 2016. MA, Mexican American; NHW, non-Hispanic white; NHB non-Hispanic black.


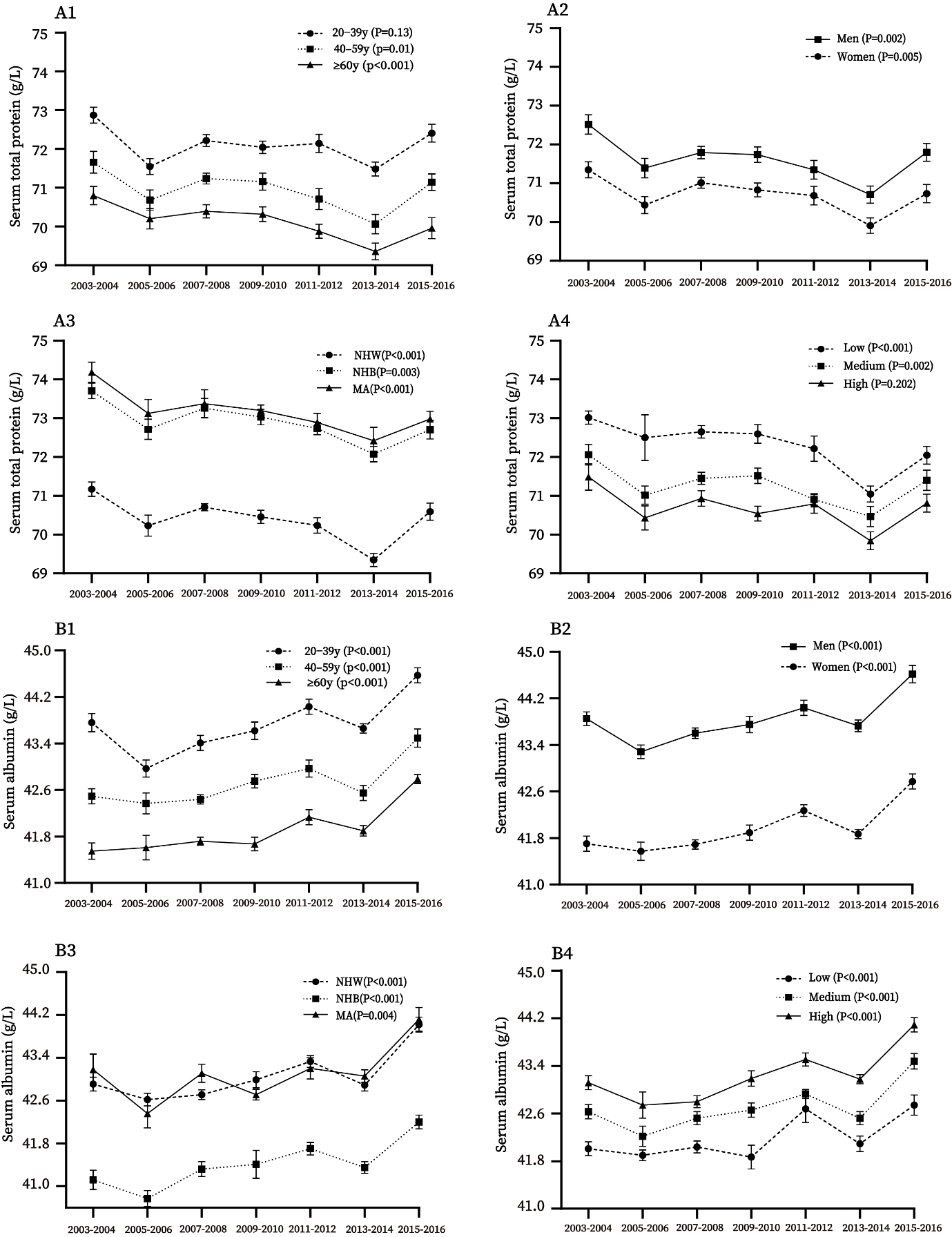


**Supplementary Figure 5** Trends of concentrations of serum total protein (A1-A4) and albumin (B1-B4) by age, sex, race-ethnicity and socioeconomic status (SS) among US adults from 2003 to 2016. MA, Mexican American; NHW, non-Hispanic white; NHB non-Hispanic black.


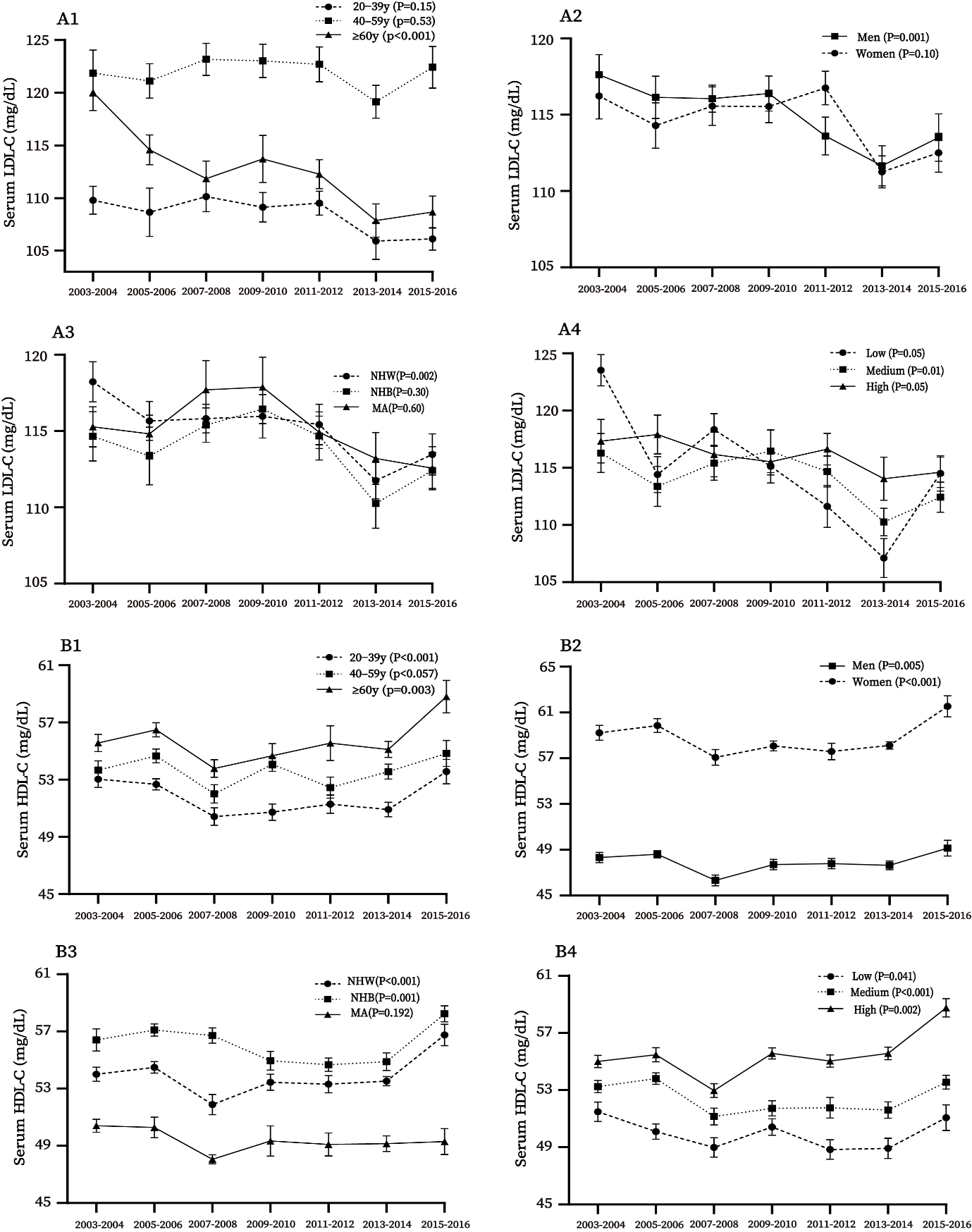


**Supplementary Figure 6** Trends of concentrations of serum total HDL-C (A1-A4) and LDL-C (B1-B4) by age, sex, race-ethnicity and socioeconomic status (SS) among US adults from 2003 to 2016. MA, Mexican American; NHW, non-Hispanic white; NHB non-Hispanic black.
